# The floral transcriptome of ylang ylang (*Cananga odorata* var. *fruticosa*) uncovers biosynthetic pathways for volatile organic compounds and a multifunctional and novel sesquiterpene synthase

**DOI:** 10.1093/jxb/erv196

**Published:** 2015-05-08

**Authors:** Jingjing Jin, Mi Jung Kim, Savitha Dhandapani, Jessica Gambino Tjhang, Jun-Lin Yin, Limsoon Wong, Rajani Sarojam, Nam-Hai Chua, In-Cheol Jang

**Affiliations:** ^1^Temasek Life Sciences Laboratory, 1 Research Link, National University of Singapore, Singapore 117604; ^2^School of Computing, National University of Singapore, Singapore 117417; ^3^Department of Biological Sciences, National University of Singapore, Singapore 117543; ^4^Laboratory of Plant Molecular Biology, The Rockefeller University, 1230 York Avenue, New York, NY 10065, USA

**Keywords:** β-Copaene, β-cubebene, β-ylangene, *Cananga odorata* var. *fruticosa*, floral scent, terpene synthase, terpenes, volatile organic compounds, ylang ylang.

## Abstract

The pleasant fragrance of ylang ylang varieties (*Cananga odorata*) is mainly due to volatile organic compounds (VOCs) produced by the flowers. Floral scents are a key factor in plant–insect interactions and are vital for successful pollination. *C. odorata* var. *fruticosa*, or dwarf ylang ylang, is a variety of ylang ylang that is popularly grown in Southeast Asia as a small shrub with aromatic flowers. Here, we describe the combined use of bioinformatics and chemical analysis to discover genes for the VOC biosynthesis pathways and related genes. The scented flowers of *C. odorata* var. *fruticosa* were analysed by gas chromatography/mass spectrometry and a total of 49 VOCs were identified at four different stages of flower development. The bulk of these VOCs were terpenes, mainly sesquiterpenes. To identify the various terpene synthases (TPSs) involved in the production of these essential oils, we performed RNA sequencing on mature flowers. From the RNA sequencing data, four full-length TPSs were functionally characterized. *In vitro* assays showed that two of these TPSs were mono-TPSs. CoTPS1 synthesized four products corresponding to β-thujene, sabinene, β-pinene, and α-terpinene from geranyl pyrophosphate and CoTPS4 produced geraniol from geranyl pyrophosphate. The other two TPSs were identified as sesqui-TPSs. CoTPS3 catalysed the conversion of farnesyl pyrophosphate to α-bergamotene, whereas CoTPS2 was found to be a multifunctional and novel TPS that could catalyse the synthesis of three sesquiterpenes, β-ylangene, β-copaene, and β-cubebene. Additionally, the activities of the two sesqui-TPSs were confirmed *in planta* by transient expression of these TPS genes in *Nicotiana benthamiana* leaves by *Agrobacterium*-mediated infiltration.

## Highlight

Combined RNA sequencing and chemical analysis led to the identification of biosynthetic pathway genes for volatile organic compounds and the discovery of novel terpene synthases in ylang ylang flowers.

## Introduction

Plants emit a large group of phytogenic volatile organic compounds (VOCs) for their defence against pathogens, parasites, and herbivores, and for attracting pollinators (Kessler and Baldwin, 2001; Dudareva *et al.*, 2013). VOCs are synthesized in all plant organs such as flowers, stems, leaves, roots, fruits and seeds, but the quantity and diversity of VOCs change in response to environmental stimuli (Dudareva *et al.*, 2013). VOCs are major components of floral scent in a wide range of flowers (Knudsen *et al.*, 1993). Over 1700 floral VOCs have been identified from 90 different plants, and they are assumed to function in both attraction of pollinators and defence against pathogens (Knudsen *et al.*, 2006; Muhlemann *et al.*, 2014). Given the important role of VOCs, their production and emission are highly regulated both spatially and developmentally.

Floral VOCs are mainly composed of terpenoids, phenylpropanoids/benzenoids, and volatile fatty acid derivatives, which are derived from different biosynthetic routes in plants (Muhlemann *et al.*, 2014). Terpenoids, also referred to as isoprenoids, are the largest and most diverse class of VOCs in plants (Dudareva *et al.*, 2013). Terpenes are synthesized from two distinct and compartmentally separated pathways, the mevalonate (MVA) and 2-C-methyl-d-erythritol 4-phosphate (MEP) pathways (McGarvey and Croteau, 1995). The phenylpropanoid and benzenoid class of metabolites is derived primarily from the carbon skeleton of phenylalanine, which is produced by the shikimate pathway (Orlova *et al.*, 2006; Vogt, 2010).

Terpene synthases (TPSs) are responsible for generating the immense diversity in terpenes produced by plants (McGarvey and Croteau, 1995). Many TPSs have the ability to synthesize multiple products from a single prenyl diphosphate substrate (Degenhardt *et al.*, 2009). Based on sequence relatedness and functional assessment, the TPS gene family has been divided into seven subfamilies designated TPS-a to TPS-g (Bohlmann *et al.*, 1998; Lee and Chappell, 2008; Martin *et al.*, 2010). The TPS-a subfamily typically contains angiosperm-specific sesqui-TPSs, whereas angiosperm mono-TPSs form the TPS-b subfamily. The TPS-b subfamily contains the arginine/tryptophan motif R(R)X_8_W, which plays a role in the RR-dependent isomerization of GPP (Martin *et al.*, 2010). Another angiosperm mono-TPS subfamily, TPS-g, contains mono-TPSs that lack the R(R)X_8_W motif. These TPSs produce acyclic monoterpenes that contribute to the floral VOCs (Dudareva *et al.*, 2013). The TPS-c and TPS-e subfamilies consist of angiosperm di-TPSs responsible for gibberellic acid biosynthesis, namely copalyl diphosphate synthases and kaurene synthases. The different mono-, sesqui-, and di-TPS genes encoding enzymes for the synthesis of conifer-specialized terpenes belong to the gymnosperm-specific TPS-d subfamily (Martin *et al.*, 2004). TPS-f includes the monoterpene linalool synthase of the genus *Clarkia* (Dudareva *et al.*, 1996).

*Cananga odorata*, commonly called ylang ylang, is a tropical evergreen tree of the family *Annonaceae* that produces fragrant flowers and is widely cultivated throughout Southeast Asia. Essential oils obtained by steam distillation from mature fresh ylang ylang flowers are used in the cosmetic industry as major components of perfumes and fragrances, in the food industry as ingredients of aromas and flavours, and in the pharmaceutical industry as active components of antibacterials and in aromatherapy (Gaydou *et al.*, 1986; Burdock and Carabin, 2008; Benini *et al.*, 2010). The chemical composition of floral VOCs produced by ylang ylang varieties has been reported previously (Gaydou *et al.*, 1986; Benini *et al.*, 2010, 2012; Brokl *et al.*, 2013). These studies showed the presence of the volatile terpenes benzenoid and phenylpropanoid in floral VOCs. Gaydou *et al.* (1986) described the composition of essential oils of ylang ylang flowers originating from Madagascar (*C. odorata* Hook Fil. et Thomson forma genuina). These authors found that the primary component was the monoterpene linalool (19%), and the other major compounds were two sesquiterpenes, β-caryophyllene (10.7%) and germacrene D (10.3%). Additionally, this variety of ylang ylang from Madagascar contained more than 20% of other aromatic compounds such as *p*-methylanisole, benzyl benzoate, methyl benzoate, and benzyl salicylate (Gaydou *et al.*, 1986). *C. odorata* var. *fruticosa*, or dwarf ylang ylang, is another variety that is popularly grown in Southeast Asia as a small, compact shrub with highly scented flowers. Its essential oil is also used in the perfume industry. Despite the economic and social importance of this species, biosynthetic pathways leading to the production of the floral scent of this ylang ylang variety are not yet fully understood.

High-throughput RNA sequencing (RNA-seq) has increasingly become the method of choice to discover genes for metabolic pathways. We determined the chemical composition of floral VOCs at four different stages of flower formation and performed RNA-seq on mature yellow flowers of *C. odorata* var. *fruticosa* where the production of floral VOCs is at the maximum. Terpenes formed the bulk of floral VOCs in this variety. Our transcriptome data revealed 16 TPS transcripts from dwarf ylang ylang flowers of which four were functionally characterized in this study.

## Materials and methods

### Plant materials

The yellow flowers of *C. odorata* var. *fruticosa* (dwarf ylang ylang) grown in Singapore were collected in November for RNA-seq. Four different stages of flowers and leaves of dwarf ylang ylang were obtained in April for further experiments. The four different stages of flowers were as follows: B, bud stage: completely closed petal, green; I, initial-flowering stage: semi-open small and short petals, green, 9 d after bud stage; II, full-flowering stage: completely open large and long petals, yellowish green, 20 d after bud stage; and III, end-flowering stage: fully matured petals, yellow, 30 d after bud stage.

Four-week-old *Nicotiana benthamiana* plants grown in a greenhouse were used for *in vivo* characterization and subcellular localization of CoTPSs.

### Extraction of essential oils from ylang ylang flowers

Flowers and leaves from ylang ylang were frozen in liquid nitrogen and ground to a powder with a pre-chilled mortar and pestle. About 500mg of powder was dissolved in 500 µl of ethyl acetate (Fisher Scientific) including 1 µl (10mg ml^–1^) of camphor (Sigma-Aldrich) as an internal standard. The slush was vortexed and incubated on a horizontal shaker at 50rpm for 2h. After centrifugation of the mixture at 13 000*g* for 10min, the resulting ethyl acetate upper layer extract was transferred to a clean Eppendorf tube and mixed with 300mg of anhydrous Na_2_SO_4_ (Sigma-Aldrich) to remove water. Following the second centrifugation, the extract was transferred into a 2ml glass vial for gas chromatography/mass spectrometry (GC-MS) analysis (Agilent Technologies).

### RNA isolation for RNA sequencing

Frozen ylang ylang flowers were homogenized using a pre-chilled mortar and pestle into a fine powder, and total RNA was isolated using the TRIzol method (Invitrogen). Purified RNA samples were first treated with RNase-free DNase I (Roche) to remove genomic DNA and then extracted using chloroform. The RNA quantity was determined with a Nanodrop spectrophotometer (ND-1000; Thermo Fisher Scientific). The RNA integrity number (RIN) was evaluated using an Agilent 2100 bioanalyzer and RNA 6000 Nano Labchip kit (Agilent Technologies). RNA with a RIN value of 7<*x*<10 was sent for RNA-seq to the Rockefeller University Genomics Resource Center (New York, USA). RNA-seq and assembly were carried out as described by Jin *et al.* (2014).

### Quantitative real-time reverse transcription PCR (qRT-PCR)

Quantitative RT-PCR was performed to investigate gene expression patterns during ylang ylang flower development. One microgram of total RNA was used for first-strand cDNA synthesis with Moloney murine leukemia virus reverse transcriptase (Promega). The qRT-PCRs were performed using an Applied Biosystems 7900HT Fast Real-time PCR System and Applied Biosystems Power SYBR Green PCR Master Mix (Life Technologies). Oligonucleotide primers for qRT-PCR were designed for selected genes of the MEP, MVA, shikimate, benzyl, and phenyl propanoid pathways using Primer3 (http://bioinfo.ut.ee/primer3-0.4.0/) and are listed in Supplementary Table S1, available at *JXB* online. Each PCR product obtained from regular PCR was cloned into the pGEM-T Easy vector (Promega) and verified by sequencing. The specificity of the amplified PCR product was assessed by melting-curve analysis. All experiments were carried out in technical triplicates and with biological duplicates. A non-template control was included for each gene to exclude random/reagent contamination and primer-dimer formation. A mock reaction containing all the RT-PCR reagents except the reverse transcriptase was used as a negative control. The *Actin* gene was used for normalization in each qRT-PCR.

### Sequence identification, multiple sequence alignment and phylogenetic analysis

DNA sequences were edited and assembled using Lasergene 8 (DNASTAR). The phylogenetic analysis of CoTPSs was performed using the maximum likelihood method in MEGA version 6 (Tamura *et al.*, 2011).

### Isolation of the full-length open reading frame (ORF) of CoTPS and vector construction for *Agrobacterium*-mediated gene expression

Full-length ORFs of *CoTPS* genes were amplified by PCR from dwarf ylang ylang flower-derived cDNA with the primer sets listed in Supplementary Table S1. Purified PCR products were cloned into pENTR/D-TOPO (Invitrogen). For yellow fluorescent protein (YFP) fusion constructs, the pTOPO clone harbouring each *CoTPS* gene was integrated into the destination vector, the pBA-DC-YFP expression vector (Zhang *et al.*, 2005), which contains the cauliflower mosaic virus (CaMV) 35S promoter and the C-terminal in-frame *YFP* gene to create *CoTPS–YFP* using LR Clonase (Invitrogen). All constructs were verified by DNA sequencing. The final plasmid was transformed into *Agrobacterium tumefaciens* GV3101 by electroporation (Bio-Rad), plated on an LB plate containing spectinomycin (100 µg ml^–1^) and gentamycin (20 µg ml^–1^), and incubated at 28 °C for 2 d.

### Subcellular localization of CoTPSs

*Agrobacterium*-mediated transient gene expression was performed using leaves of 4-week-old *N. benthamiana* plant as described by Jin *et al.* (2014). Infiltrated *N. benthamiana* leaves expressing YFP-fused protein were excised, mounted on slides, and imaged using a confocal laser-scanning microscope (Carl Zeiss LSM5 Exciter) with a standard filter set. Images were processed with the LSM Image Browser (Carl Zeiss).

### Preparation of recombinant proteins

To construct vectors for the recombinant N-terminal poly-histidine (6His)-tagged proteins, the full-length ORF of *CoTPS* was amplified by PCR with primers designed with restriction enzymes sites at the ends (Supplementary Table S1). The PCR-amplified product and pET28b plasmid (Novagen) were digested separately with the corresponding restriction enzymes (New England Biolabs) and purified using a Qiagen PCR Purification kit. The digested PCR product was then cloned into the pET28b expression vector using a Rapid DNA Ligation kit (Roche). The final construct was transformed into *Escherichia coli* BL21(DE3)pLysS (Invitrogen), and recombinant proteins were purified from *E.coli* extracts after isopropyl β-d-1-thiogalactopyranoside induction as described previously (Jang *et al.*, 2005).

### *In vitro* TPS assay

An *in vitro* enzyme assay for TPS activity was performed in a final volume of 500 μl of reaction buffer [25mM HEPES, pH 7.4, 100mM KCl, 7.5mM MgCl_2_, 5% (v/v) glycerol, 5mM dithiothreitol], with about 20 μg of recombinant protein and 10 µg of either farnesyl pyrophosphate (FPP) or geranyl pyrophosphate (GPP) (Sigma-Aldrich). The reaction mixtures were mixed gently and carefully overlaid with 250 µl of hexane (Sigma-Aldrich) to trap volatile products. The tube was then sealed with Parafilm and incubated at 30 °C for 2h, followed by 1min of vortexing. After centrifugation at 1200*g* at 4 °C for 30min, the hexane upper layer was transferred into a 2ml glass vial for GC-MS analysis (see below). As a negative control, heat-inactivated recombinant protein was added to the enzyme assay.

### *In vivo* characterization of CoTPSs

The underside of *N. benthamiana* leaves was infiltrated with an *Agrobacterium* strain harbouring the *CoTPS* construct with or without the strain carrying *Arabidopsis* 3-hydroxy-3-methylglutaryl-CoA reductase (*AtHMGR*) under the control of a CaMV 35S promoter (Jin *et al.*, 2014). All experiments were carried out with co-expression of the viral-encoded protein P19, which improves transgene expression by suppressing post-transcriptional gene silencing (Voinnet *et al.*, 2003). After infiltration, the tobacco plants were maintained in a growth chamber at 25 °C, under long-day conditions (16h light/8h dark) for 3 d. Four to five infiltrated leaves were then frozen immediately in liquid nitrogen and homogenized with a pre-chilled mortar and pestle. Up to 400–600mg of leaf powder was obtained from four to five leaves. Subsequent sample processing for GC-MS analysis was performed as described above. A *CaMV35S::AtHMGR* construct served as a negative control.

### GC-MS analysis

GC-MS analysis was performed on an Agilent 7890A GC system and an Agilent Technologies 5975C Inert XL Mass Selective Detector, equipped with an HP-5MS UI column (30 m×0.25 mm×0.25 μm; Agilent Technologies). Conditions were as follows: 5 µl sample injection, splitless injection, oven program 50 °C (1min hold) at 8 °C min^–1^ to 300 °C (5min hold). For data processing, MSD ChemStation Data Analysis (Agilent Technologies) was used. The essential oil components were identified by comparison of their mass spectra with those in the NIST 2011 library data for GC-MS and comparison of their retention indices (RIs). The RIs were determined on the basis of an *n*-alkanes (C8–C40) mix standard (Sigma-Aldrich) under the same operation conditions. Camphor was added to serve as an internal standard. The amount of each compound was calculated by measuring its peak area related to that of a known amount of camphor. The identified components along with their RIs and relative percentage values are listed in Table 1.

**Table 1.** Essential oils composition of the flowers from C. odorata var. fruticosa

					RA (%)^*d*^			
No.^*a*^	Compounds	RT (min)^*b*^	RI^*c*^	Formula	Bud	I	II	III
1	β-Thujene	9.507	904	C_10_H_16_	–	–	0.21	0.36
2	α-Pinene	9.652	919	C_10_H_16_	3.08	1.30	0.99	0.65
3	Camphene	9.894	943	C_10_H_16_	–	–	–	0.13
4	Sabinene	10.199	974	C_10_H_16_	–	–	0.26	0.43
5	β-Pinene	10.342	988	C_10_H_16_	–	–	0.45	0.66
6	α-Phellandrene	10.661	993	C_10_H_16_	–	–	–	0.05
7	α-Terpinene	10.846	1012	C_10_H_16_	–	–	–	0.27
8	p-Cresol methyl ether	10.884	1016	C_8_H_10_O	–	–	–	0.49
9	*trans*-β-Ocimene	11.054	1023	C_10_H_16_	–	0.46	0.88	1.55
10	β-Ocimene	11.170	1024	C_10_H_16_	–	0.40	1.09	1.99
11	γ-Terpinene	11.397	1027	C_10_H_16_	–	–	–	0.15
12	Terpinolene	12.011	1032	C_10_H_16_	–	–	–	0.16
13	β-Linalool	12.102	1040	C_10_H_18_O	–	0.38	0.99	1.40
14	Neo-allo-ocimene	12.594	1089	C_10_H_16_	–	–	–	0.18
15	3,4-Dimethoxytoluene	14.460	1197	C_9_H_12_O_2_	–	–	–	0.39
16	2-Methoxy-4-vinylphenol	15.854	1276	C_9_H_10_O_2_	–	–	–	0.19
17	γ-Elemene	16.176	1306	C_15_H_24_	–	–	–	0.16
18	Eugenol	16.481	1309	C_10_H_12_O_2_	–	–	–	0.17
19	α-Copaene	16.831	1321	C_15_H_24_	1.81	1.24	0.83	0.44
20	γ-Gurjunene	17.025	1340	C_15_H_24_	3.00	1.16	0.56	0.32
21	Methyleugenol	17.062	1344	C_11_H_14_O_2_	–	–	–	0.08
22	β-Caryophyllene	17.600	1398	C_15_H_24_	16.47	23.50	15.90	11.57
23	β-Ylangene	17.683	1406	C_15_H_24_	3.55	1.80	1.16	0.63
24	(E)-β-Farnesene	17.836	1421	C_15_H_24_	–	0.51	0.61	0.77
25	γ-Muurolene	17.910	1429	C_15_H_24_	1.21	0.60	0.38	0.20
26	Humulene	18.020	1440	C_15_H_24_	3.44	3.44	2.24	1.63
27	β-Cubebene	18.110	1449	C_15_H_24_	1.02	0.35	0.19	0.08
28	GermacreneD	18.580	1450	C_15_H_24_	50.17	33.17	27.33	13.26
29	α-Farnesene	18.686	1461	C_15_H_24_	–	10.03	19.89	31.50
30	α-Bergamotene	18.771	1469	C_15_H_24_	–	18.67	23.63	26.79
31	Cedrene	18.932	1485	C_15_H_24_	–	–	0.32	0.33
32	δ-Cadinene	18.989	1491	C_15_H_24_	1.54	0.43	0.17	0.17
33	α-Patchoulene	19.035	1495	C_15_H_24_	–	–	0.07	0.13
34	Elemicin	19.233	1515	C_12_H_16_O_3_	–	–	–	0.13
35	Germacrene D-4-ol	19.845	1537	C_15_H_26_O	0.75	0.21	0.16	0.13
36	β-Caryophyllene oxide	19.950	1548	C_15_H_24_O	–	–	–	0.07
37	Isoelemicin	20.561	1609	C_12_H_16_O_3_	–	–	0.15	0.13
38	Farnesol	21.527	1763	C_15_H_26_O	–	0.88	0.85	0.75
39	Benzyl benzoate	22.236	1812	C_14_H_12_O_2_	–	–	0.27	0.69
40	*cis*-11-Hexadecenal	22.505	1830	C_16_H_30_O	–	–	–	0.11
41	Octadecanal	22.623	1841	C_18_H_36_O	–	–	–	0.07
42	(E,E,) farnesol acetate	22.941	1873	C_17_H_28_O_2_	–	–	0.10	0.10
43	Z-9-Hexadecen-1-ol	23.328	1912	C_16_H_32_O	–	–	–	0.17
44	9-Nonadecene	23.475	1918	C_19_H_38_	–	–	–	0.18
45	Benzyl salicylate	23.600	1931	C_14_H_12_O_3_	–	–	–	0.21
46	δ-EIemene	16.173	1308	C_15_H_24_	4.81	0.98	0.34	–
47	α-Ylangene	18.662	1458	C_15_H_24_	7.16	–	–	–
48	γ-Cadinene	18.910	1483	C_15_H_24_	1.31	0.52	–	–
49	α-Copaene-11-ol	21.094	1720	C_15_H_24_O	0.67	–	–	–

^*a*^ Compounds listed in order of elution from a HP-5MS UI column.

^*b*^ RT, retention time (min).

^*c*^ RI, retention indices calculated against C_8_–C_40_ *n*-alkanes on the HP-5MS UI column.

^*d*^ RA, relative amount (%); ratio expressed against the sum of all peaks.

### Accession number

The RNA-seq data supporting this study are available in the DNA Data Bank of Japan (DDBJ: http://www.ddbj.nig.ac.jp/) under accession number DRA002822.

## Results

### Stage-specific variations of VOCs in dwarf ylang ylang flowers

**Fig. 1.**
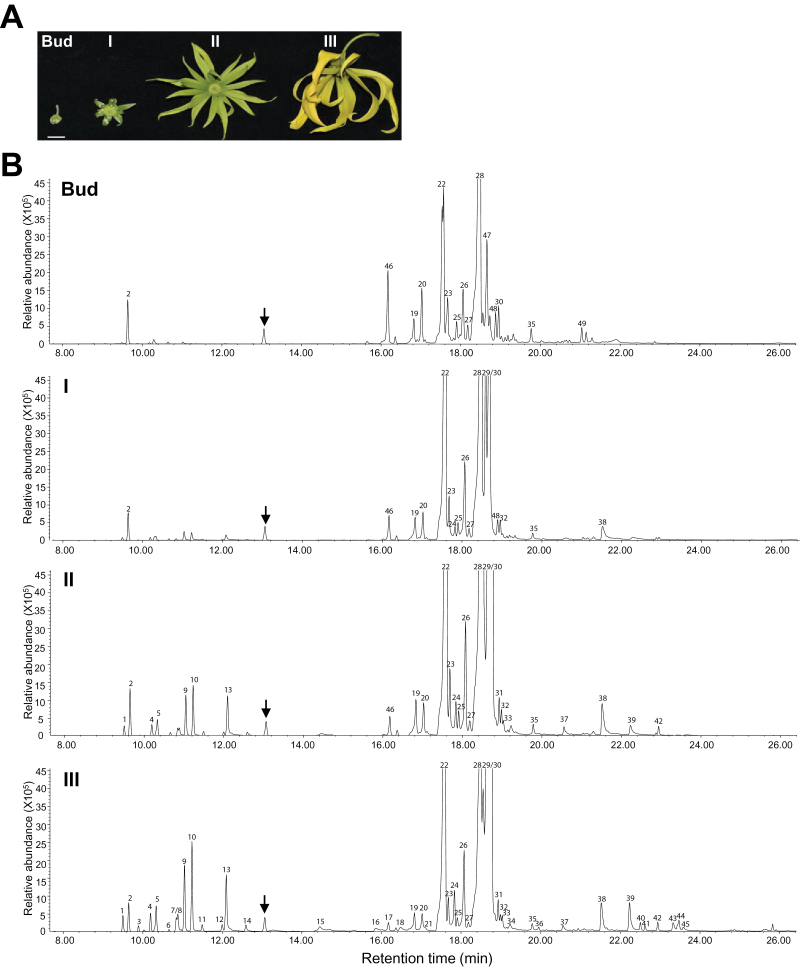
Compositional variation of dwarf ylang ylang essential oils during flower development. (A) Photograph of dwarf ylang ylang flowers showing the development of a newly emerged flower bud to a fully open yellow flower. I, Undeveloped small flower; II, mature green flower; III, fully mature yellow flower. Bar, 1cm. (B) GC traces of essential oils from floral buds, undeveloped small flowers (I), mature green flowers (II), and fully mature yellow flowers (III). The arrows indicate the peak of camphor (10 µg µl^–1^), the internal standard used in the assay. The peaks numbered in the traces are identical to those listed in Table 1. The traces are magnified images of the corresponding zones indicated by a dotted line in Supplementary Fig. S1, available at *JXB* online. (This figure is available in colour at *JXB* online.)

Flowers can emit different volatile compounds at different stages of development (Dudareva *et al.*, 2000). Dwarf ylang ylang flowers have little floral scent when the petals are green, but their scent gradually becomes stronger as the flower matures. To examine the overall intensity and the diversity of the floral scent during flower development, total essential oils from flowers at four different stages of development were analysed by GC-MS (Fig. 1). Figure 1B and Supplementary Fig. S1 show that the chemical composition of the essential oils from the floral bud stage to the three different stages of open flower development was very diverse, both quantitatively and qualitatively. Only 15 compounds that had meaningful levels of >0.1% of total volatile compounds were detected from floral buds (Fig. 1B, Table 1). The number of peaks increased progressively during maturation of flower buds into fully open flowers. More than 20, 27, and 45 volatile compounds were obtained from the three different stages of flower development: undeveloped small flower (I), mature green flower (II), and fully mature yellow flower (III). Hence, the fully mature stage represents the stage where there was maximum production of VOCs by the flowers. At this stage, the majority of the volatiles were terpenes with a few benzenoid/phenolpropanoid compounds. Out of 45 compounds identified, 31 were identified as mono- and sesquiterpenes using the mass spectra reference library (Fig. 1B and Table 1). Interestingly, >90% of the total identified terpenes were sesquiterpenes consisting of α-farnesene (31.50%), α-bergamotene (26.79%), germacrene D (13.26%), β-caryophyllene (11.57%), humulene (1.63%), farnesol (0.75%), *trans*-β-farnesene (0.77%), and β-ylangen (0.63%), whereas monoterpenes were quantitatively less than 10%, comprising mainly *cis*-β-ocimene (1.99%), *trans*-β-ocimene (1.55%), and β-linalool (1.40%). Other aromatic compounds comprised <3%. The relative amounts of all identified volatiles are shown in Table 1 (see mature yellow flowers, III). Dried yellow flowers of ylang ylang also showed a similar volatile composition (Supplementary Fig. S2A, available at *JXB* online). Compared with flowers, ylang ylang leaves contained very low levels of terpenes, comprising mainly α-pinene, β-caryophyllene, germacrene D, and phytol (Supplementary Fig. S2B).

Two sesquiterpenes, germacrene D and β-caryophyllene, could be found at all stages of development, and their levels were retained or slightly decreased during open flower development (#22 and #28, Fig. 1B, and Supplementary Fig. S3, available at *JXB* online). However, the other two major sesquiterpenes, α-farnesene and α-bergamotene, were undetectable at the floral bud stage but were found during early flower development (#29 and #30 in I, Fig. 1B, and Supplementary Fig. S3) and subsequently became the most abundant components of essential oils at mature stages of flower development (II and III, Fig. 1B). Interestingly, most of the monoterpenes except α-pinene were undetectable at the floral bud stage, but they gradually increased during open flower development (Fig. 1B). Among the monoterpenes, *trans*-β-ocimene, β-ocimene, and β-linalool were highly inducible during flower maturation (#9, #10, and #13, Fig. 1B). Additionally, our GC-MS analysis identified several sesquiterpenes or sesquiterpene alcohols that decreased during flower maturation such as δ-elemene (#46), α-ylangene (#47), γ-cadinene (#48), and α-copaene-11-ol (#49). Other aromatic compounds such as benzenoid/phenolpropanoid and volatile fatty acids were found almost exclusively in mature yellow flowers (Fig. 1B and Supplementary Fig. S1, Table 1). GC-MS analysis of flowers at night did not show any change in VOC profile, suggesting that there are no significant diurnal changes in the emission pattern (Supplementary Fig. S4, available at *JXB* online).

### RNA sequencing, *de novo* assembly, and annotation of the transcriptome

**Fig. 2.**
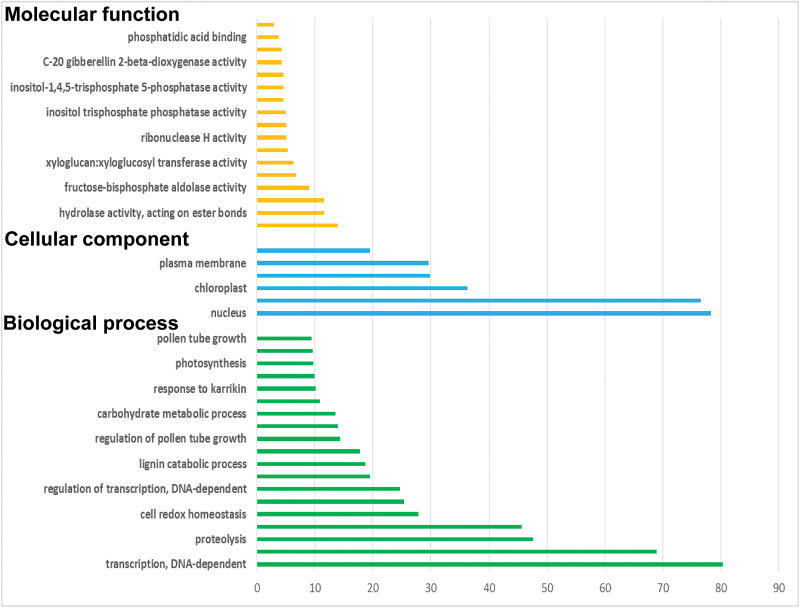
GO terms for the top 1000 highly expressed transcripts in dwarf ylang ylang flowers. (This figure is available in colour at *JXB* online.)

To profile the dwarf ylang ylang floral transcriptome, we sequenced RNA-seq libraries synthesized from the mature yellow flowers. Illumina sequencing runs generated more than 110 million reads of 101bp, and the quality of reads was evaluated by FastQC (http://www.bioinformatics.babraham.ac.uk/projects/fastqc/) (Supplementary Fig. S5, available at *JXB* online). Due to the absence of reference genomic sequences for ylang ylang, the Trinity method was used for *de novo* assembly of short sequence reads (Grabherr *et al.*, 2011). These assemblies generated a total of 45 379 unigenes with an N50 value of 2016bp (Table 2). The assembled unigenes were BLASTed against the National Centre for Biotechnology Information non-redundant (nr) protein database and protein databases for *Arabidopsis thaliana*, *Vitis vinifera*, and *Oryza sativa*. Among 45 379 unigenes, 30 539 unigenes (67.3%) were annotated through a BlastX search with E-values ≤1e–3 (Table 2). Functional classifications of Gene Ontology (GO) terms of all unigenes were performed using Trinotate (Quevillon *et al.*, 2005). Figure 2 shows enriched GO terms for the top 1000 highly expressed transcripts. From our annotated unigenes, 16 were identified as TPSs that were more than 500bp. Of these, four unigenes contained full-length ORFs encoding TPS.

**Table 2.** Overview of the assembly results of RNA-seq

No. isoforms	N50 (bp)	No. unigenes	No. annotated	% Annotation
86 512	2016	45 379	30 539	67.3

### RNA-seq analysis of different biosynthetic pathways active in flowers

GC-MS analysis of the mature flowers showed that the majority of VOCs were terpenes. We used our RNA-seq data to analyse the expression profile of genes encoding enzymes for the precursor pathways leading to the formation of VOCs in mature flowers. The majority of ylang ylang orthologue unigenes were full length and showed high sequence similarity to known enzymes of these pathways from other plants (Supplementary Fig. S6, available at *JXB* online).

**Fig. 3.**
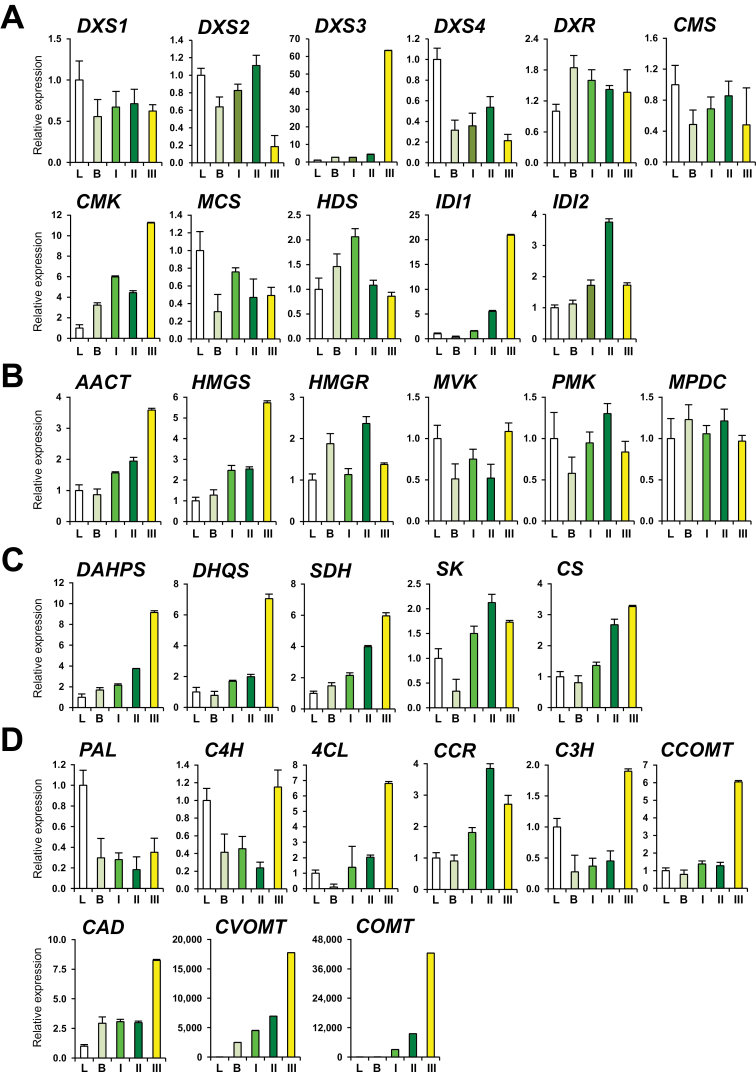
qRT-PCR analyses of different biosynthetic pathway genes. Expression of genes involved in the MEP (A), MVA (B), shikimate (C), and phenyl propanoid (D) biosynthetic pathways were examined from leaves (L), buds (B), and three different stages of flower development: undeveloped small flowers (I), mature green flowers (II), and fully mature yellow flowers (III) by qRT- PCR. DXS, 1-deoxy-d-xylulose 5- phosphate synthase; DXR, 1-deoxy-d-xylulose 5-phosphate reductoisomerase; CMS, 2-C-methyl-d-erythritol 4-phosphate cytidylyltransferase; CMK, 4-(cytidine 5′-diphospho)-2-C-methyl-d-erythritol kinase; MCS, 2-C-methyl-d-erythritol 2,4-cyclodiphosphate synthase; HDS, 4- hydroxy-3-methylbut-2-en-1-yl diphosphate synthase; IDI, isopentenyl pyrophosphate isomerase; AACT, acetyl-CoA acetyltransferase; HMGS, hydroxymethylglutaryl-CoA synthase; HMGR, hydroxymethylglutaryl-CoA reductase; MVK, mevalonate kinase; PMK, phosphomevalonate kinase; MPDC, mevalonate diphosphate decarboxylase; DAHPS, 3-deoxy-d-arabino-heptulosonate-7-phosphate synthase; DHQS, 3-dehydroquinate synthase; SDH, shikimate dehydrogenase; SK, shikimate kinase; CS, chorismate synthase; PAL, phenylalanine ammonia lyase; C4H, cinnamate-4-hydroxylase; 4CL, 4-coumaroyl-CoA ligase; CCR, cinnamoyl-CoA reductase; C3H, *p*-coumarate-3-hydroxylase; CCOMT, caffeoyl-CoA 3-*O*-methyltransferase; CAD, cinnamyl alcohol dehydrogenase; CVOMT, chavicol *O*-methyltransferase; COMT, caffeic acid/5-hydroxyferulic acid *O*-methyltransferase. (This figure is available in colour at *JXB* online.)

The MEP and MVA pathways are the pathways leading to the formation of mono- and sesquiterpenes. Transcripts of all the enzyme genes involved in these two pathways were detected in our RNA-seq data, and their expression was validated by qRT-PCR (Fig. 3). Additionally, the expression of these enzyme genes was also examined at earlier stages of flower development and in leaves by qRT-PCR. Genes encoding the MEP and MVA enzymes were active in all stages of flower development consistent with the high production of terpenes in the flowers (Fig. 3A, B). It has been reported that 1-deoxy-d-xylulose-5-phosphate synthase (DXS), the first enzyme of the MEP pathway, is important for the overall regulation of the pathway and is encoded by a small gene family (Cordoba *et al.*, 2011). From our RNA-seq data, we were able to identify four different *DXS* unigenes showing different levels of abundance in flowers and leaf. One of these, *DXS3*, belonging to clade 2, which may be related to secondary metabolism (Walter *et al.*, 2002; Phillips *et al.*, 2007), was highly induced in stage III flowers (Fig. 3A and Supplementary Figs S7 and Fig. S8, available at *JXB* online). The majority of the genes, except phenylalanine ammonia lyase (*PAL*), for the shikimate pathway enzymes, which produce phenylalanine for the production of benzonoids and phenylproponids, appeared to be expressed more in mature flowers (Fig. 3C, D). The significant benzenoids in VOCs were benzyl benzoate and benzyl salicylate, and the phenylproponoid was eugenol. These compounds were detected only in mature flowers (#18, #39, and #45, Fig. 1B, Table 1). Previous *in vitro* experiments have indicated that benzyl benzoate might play a role in pollinator attraction (Hoballah *et al.*, 2005; Huber *et al.*, 2005) or in plant defence (miticide) (Harju *et al.*, 2004).

### Phylogenetic analysis of *TPS* genes from dwarf ylang ylang flowers

**Fig. 4.**
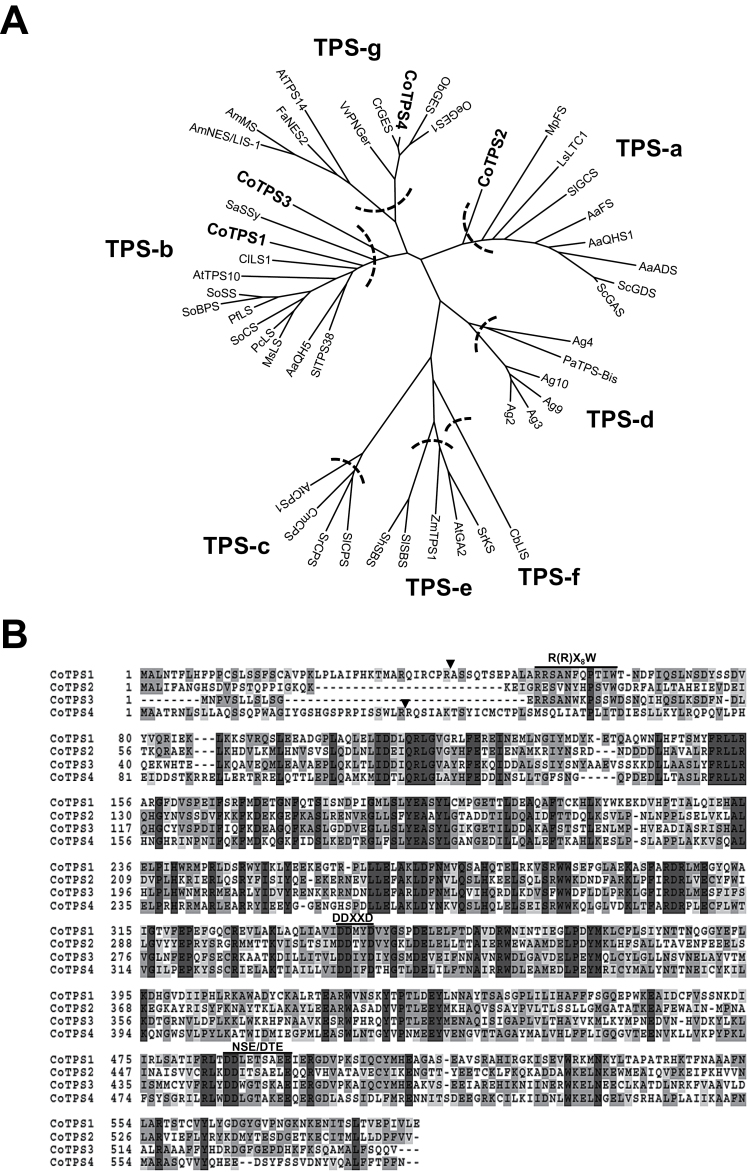
Phylogenetic analysis and alignment of TPSs from dwarf ylang ylang. (A) A maximum likelihood tree was drawn using the MEGA 6 program from an alignment of full-length CoTPSs with other plant TPSs. The seven TPS subfamilies a–g are delimited by dashed lines, based on the taxonomic distribution of the TPS families (Chen *et al.*, 2011). AaADS, *Artemisia annua* (Aa) amorpha-4,11-diene synthase (NCBI Protein no. AFA34434); AaFS, β-farnesene synthase (Q9FXY7); AaQHS1, β-caryophyllene synthase (AAL79181); AaQH5, linalool synthase (AAF13356); Ag10, *Abies grandis* (Ag) 4S-limonene synthase (AAB70907); Ag2, myrcene synthase (AAB71084); Ag4, δ-selinene synthase (AAC05727); Ag3, pinene synthase (AAB71085); Ag9, terpinolene synthase (AAF61454); AmNES/LIS-1, *Antirrhium majus* (Am) nerolidol/linalool synthase1 (ABR24417); AmMS, myrcene synthase (AAO41727); AtCPS1, *Arabidopsis thaliana* (At) copalyl diphosphate synthase (NP_192187); AtGA2, kaurene synthase; AtTPS10 (AAC39443), myrcene/ocimene synthase (AAG09310); AtTPS14, linalool synthase (NP176361); CbLIS, *Clarkia breweri S*-linalool synthase (AAC49395); ClLS1, *Citrus limon* limonene synthase 1 (AAM53944); CmCPS, *Cucurbits maxima* copalyl diphosphate synthase (AAD04292); CrGES, *Catharanthus roseus* geraniol synthase (AFD64744); FaNES2, *Fragaria*×*ananassa* nerolidol synthase (CAD57081); LsLTC1, *Lactuca sativus* germacrene A synthase (AAM11626); MpFS, *Mentha piperita* β-farnesene synthase (AAB95209); MsLS, *Mentha spicata* 4S-limonene synthase (AAC37366); ObGES, *Ocimum basilicum* geraniol synthase (AAR11765); OeGES1, *Olea europaea* geraniol synthase 1 (AFI47926); PaTPS-Bis, *Picea abies* α-bisabolene synthase (AAS47689); PcLS, *Perilla citriodora* limonene synthase (AAG31435); PfLS, *Perilla frutescens* linalool synthase (AAL38029); SaSSy, *Santalum album* santalene/bergamotene synthase (ADO87000); ScGAS, *Solidago canadensis* (Sc) germacrene A synthase (CAC36896); ScGDS, germacrene D synthase (AAR31145); ShSBS, *Solanum habrochaites* santalene/bergamotene synthase (B8XA41); SlSBS, *Solanum lycopersicum* (Sl) santalene and bergamotene synthase (XP004244438); SlCPS, copalyl diphosphate synthase (BAA84918); SlGCS, germacrene C synthase (AAC39432); santalene/bergamotene synthase (BAA84918); Sl TPS38, Sl terpene synthase 38 (AEP82768); SoBPS, *Salvia officinalis* (So) bornyl diphosphate synthase (AAC26017); SoCS, 1,8-cineole synthase (AAC26016); SoSS, sabinene synthase (AAC26018); SrCPS, *Stevia rebaudiana* (Sr) copalyl pyrophosphate synthase (AAB87091); SrKS, kaurene synthase (AAD34294); VvPNGer, *Vitis vinifera* geraniol synthase (ADR74218); ZmTPS1, *Zea mays* terpene synthase 1 (AAO18435). (B) Comparison of deduced amino acid sequences of dwarf ylang ylang TPSs. The deduced amino acid sequences of *CoTPS* genes were aligned using CLUSTAL W. The Asp-rich domain DDXXD, the R(R)X_8_W motif, and the NSE/DTE motif, which are highly conserved in plant TPSs and required for TPS activity, are indicated. Arrowheads denote the predicted cleavage sites of plastidial transit peptides. Completely conserved residues are shaded in dark grey, identical residues in grey, and similar residues in light grey. Dashes indicate gaps introduced to maximize sequence alignment.

Terpenes were the major VOCs of dwarf ylang ylang flowers. From the dwarf ylang ylang RNA-seq data, four full-length ORFs of *TPS* genes were PCR amplified from cDNA pools of the flowers. Phylogenetic analysis based on the deduced amino acid sequences of four *CoTPS* cDNAs showed that CoTPS2 (561 aa) belonged to the TPS-a subfamily representing the sesqui-TPSs, whereas CoTPS1 (590 aa) and CoTPS3 (547 aa) fell into the TPS-b subfamily, which consists mainly of mono-TPSs (Chen *et al.*, 2011; Fig. 4A). CoTPS4 (586 aa) is a member of the TPS-g subfamily, which lacks the R(R)X_8_W motif in the N-terminal region of mono-TPSs and produces acyclic monoterpenes (Dudareva *et al.*, 2003; Yuan *et al.*, 2008; Chen *et al.*, 2011; Fig. 4A). All four CoTPSs had the conserved aspartate-rich motif (DDXXD) and NSE/DTE motifs that chelate divalent metal ions, typically Mg^2+^, in the C-terminal domain (Fig. 4B). Both motifs are required for cyclization of the universal acyclic terpene precursors, such as GPP and FPP, to synthesize mono- and sesqui-terpene, respectively (Chen *et al.*, 2011). The arginine-tryptophan motif, R(R)X_8_W, present at the N terminus of most mono-TPS and in some sesqui-TPS and di-TPS, was found in CoTPS1, CoTPS2, and CoTPS3 but not in CoTPS4 (Fig. 4B). One of the distinguishing structural features between mono- and sesqui-TPS is the presence of an N-terminal plastid transit peptide (Tp) sequence. Using the signal sequence analysis programs ChloroP (http://www.cbs.dtu.dk/services/ChloroP) and WoLF PSORT (http://www.genscript.com/psort/wolf_psort.html), a putative N-terminal plastid Tp sequence of 41 and 35 aa for CoTPS1 and CoTPS4, respectively, was predicted, indicating they are likely to be mono-TPSs. However, we could not find a putative plastid Tp sequence for CoTPS3, which was supposed to be a mono-TPS belonging to the TPS-b subfamily. CoTPS2 did not contain a plastid Tp sequence, which correlated well with the prediction of it being a sesqui-TPS.

The amino acid sequences of CoTPS1 and CoTPS2 had highest identity with the magnolia (*Magnolia grandiflora*) TPSs Mg17 (70% similarity, 54% identity) and Mg25 (72% similarity, 55% identity) for α-terpineol and β-cubebene, respectively (Supplementary Fig. S9A, B, available at *JXB* online) (Lee and Chappell, 2008). CoTPS3 showed 65% amino acid similarity and 47% identity with mountain pepper (*Litsea cubeba*) *trans*-ocimene synthase (Supplementary Fig. S9C) (Chang and Chu, 2011). CoTPS4 was most similar to geraniol synthase from Madagascar periwinkle (*Catharanthus roseus*) (92% similarity, 84% identity; Supplementary Fig. S9D) (Simkin *et al.*, 2013).

### Subcellular localization and expression of the four CoTPSs

**Fig. 5.**
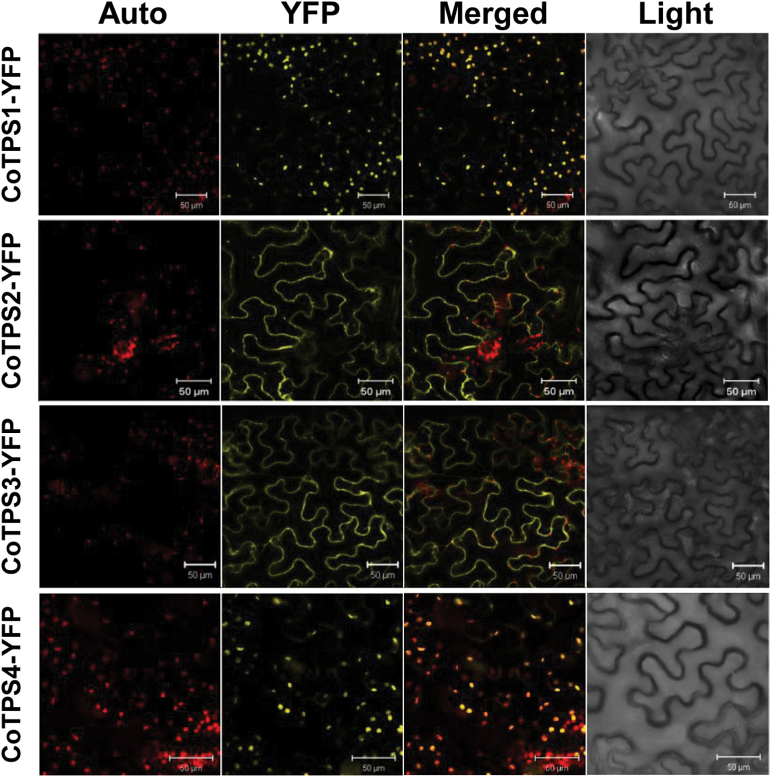
Subcellular localization of CoTPSs. YFP-fused CoTPSs (CoTPS1–YFP, CoTPS2–YFP, CoTPS3–YFP, and CoTPS4–YFP) were transiently expressed in *N. benthamiana* leaves by *Agrobacterium*-mediated infiltration and visualized at 3 d post-infiltration using the YFP channel of a confocal microscope. Auto, chlorophyll autofluorescence; YFP, YFP channel image; Light, light microscopy image; Merged, merged image between Auto and YFP. Bars, 50 µm. (This figure is available in colour at *JXB* online.)

As well as phylogenetic analysis and bioinformatics-based attempts to classify TPSs, their subcellular localization is also important for function prediction. This was especially true for CoTPS3, since its function was unpredictable from the bioinformatics analyses based on amino acid sequences. To address this issue, we transiently expressed the full-length ORF of each CoTPS fused to a YFP reporter gene to produce a CoTPS–YFP fusion protein in *N. benthamiana* leaves using *Agrobacterium*-mediated infiltration. Figure 5 shows that CoTPS1–YFP and CoTPS4–YFP, which had the N-terminal plastid Tp sequence, were localized in chloroplasts as expected, whereas CoTPS2–YFP and CoTPS3–YFP were distributed throughout the cytosol. Based on the results of the subcellular localization experiments, it is likely that CoTPS1 and CoTPS4 are involved in monoterpene synthesis in plastids, whereas CoTPS2 and CoTPS3 might produce sesquiterpenes in the cytosol.

**Fig. 6.**
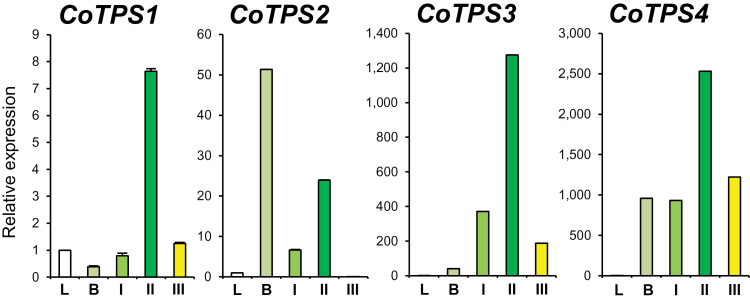
Transcript levels of dwarf ylang ylang *TPS* genes *CoTPS1*, *CoTPS2*, *CoTPS3*, and *CoTPS4* during flower development. Total RNA was isolated from leaves (L), floral buds (B), and three different stages of flower development, undeveloped small flowers (I), mature green flowers (II) and fully mature yellow flowers (III), and used as template for qRT- PCR. Amplification of *Actin* mRNA was used as an internal control. (This figure is available in colour at *JXB* online.)

The transcript levels for the four *CoTPS* genes at different developmental stages of dwarf ylang ylang flowers were examined by qRT-PCR. The expression levels of all four transcripts were very low or below detection limits in leaf tissues but greatly elevated in flower tissues (Fig. 6). Transcripts for three *TPS* genes, *CoTPS1*, *CoTPS3*, and *CoTPS4*, were highest in mature green flowers (II), whereas *CoTPS2* was highly expressed at the floral bud stage (Fig. 6).

### Functional characterization of CoTPSs

**Fig. 7.**
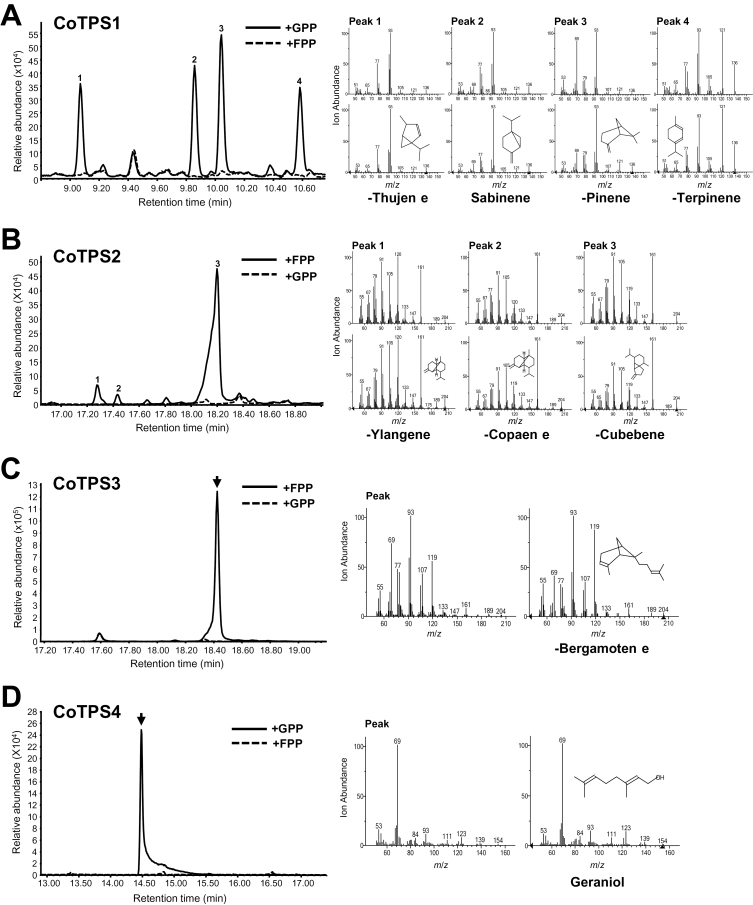
*In vitro* enzymatic assays of recombinant CoTPSs. *In vitro* enzyme assays of recombinant 6His-tagged CoTPS1 (A), CoTPS2 (B), CoTPS3 (C), or CoTPS4 (D) protein using GPP or FPP as substrate. The reaction products were analysed by GC-MS. The peaks marked with numbers or an arrow in the GC traces were identified by the mass spectra reference library. Mass spectra for the peaks formed with FPP or GPP are shown on the right side of the figure with the references. *m*/*z*, Mass-to-charge ratio.

The subcellular localization of each CoTPS–YFP fusion protein provided us with preliminary evidence to elucidate the function of each TPS. The exact functional annotation of a new TPS requires activity characterization of the recombinant protein. To determine the enzymatic activity of CoTPSs *in vitro*, 6His-tagged CoTPS recombinant proteins purified from *E. coli* BL21(DE3) (Supplementary Fig. S10, available at *JXB* online) were used for *in vitro* assays. GPP (C10) or FPP (C15) was used as the common substrate for mono- and sesqui-TPS, respectively. Control assays using heat-inactivated recombinant 6His-tagged CoTPSs did not form any terpenes from GPP or FPP (Supplementary Fig. S11, available at *JXB* online). Figure 7A shows that CoTPS1, a member of the TPS-b family, synthesized four products corresponding to β-thujene, sabinene, β-pinene, and α-terpinene from GPP, but not from FPP, which were found in the essential oil profiles of dwarf ylang ylang flowers (#1, #4, #5 and #7, Fig. 1, Table 1). These results suggested that CoTPS1 is a multifunctional β-thujene/sabinene/β-pinene/α-terpinene synthase that is able to catalyse the synthesis of a mixture of monoterpenes, namely β-thujene, sabinene, β-pinene, and α-terpinene. This is not surprising, as several multiproduct mono-TPSs that produce similar compounds, such as α-thujene, sabinene, α/β-pinene, α/γ-terpinene, have been reported widely in other plant species (Lücker *et al.*, 2002; Chen *et al.*, 2003; Fäldt *et al.*, 2003; Shimada *et al.*, 2004; Fähnrich *et al.*, 2011).

Similarly, recombinant CoTPS2 catalysed the synthesis of three compounds, β-ylangene, β-copaene, and β-cubebene, from FPP. CoTPS2 is a multifunctional β-ylangene/β-copaene/β-cubebene synthase capable of producing three sesquiterpenes, β-ylangene, β-copaene, and β-cubebene (Fig. 7B). Many sesqui-TPSs are also known to be multifunctional (Steele *et al.*, 1998; Lee and Chappell, 2008). However, TPSs that produce β-ylangene/β-copaene/β-cubebene have not yet been reported. Of these three sesquiterpene compounds, we could only detect β-ylangene and β-cubebene in the flowers of dwarf ylang ylang (#23 and #27, Fig. 1, Table 1).

CoTPS3 is a member of the TPS-b family with the unusual feature that it lacks a putative N-terminal Tp sequence. Our enzyme assays showed that CoTPS3 catalysed the formation of α-bergamotene from FPP (Fig. 7C), which is a major sesquiterpene produced in the flowers of ylang ylang (#30, Fig. 1, Table 1). CoTPS4, which belongs to TPS-g family, was capable of utilizing GPP to synthesize an acyclic monoterpene, geraniol (Fig. 7D). This was confirmed by comparison of retention time and mass spectra with those of an authentic standard (Supplementary Fig. S11). This result was expected, as the protein showed the highest amino acid identity with geraniol synthases (84%) from Madagascar periwinkle (Supplementary Fig. S9D) (Simkin *et al.*, 2013). However, geraniol was not detected in our GC-MS analysis of ylang ylang flowers.

### Functional characterization of CoTPS *in vivo*

**Fig. 8.**
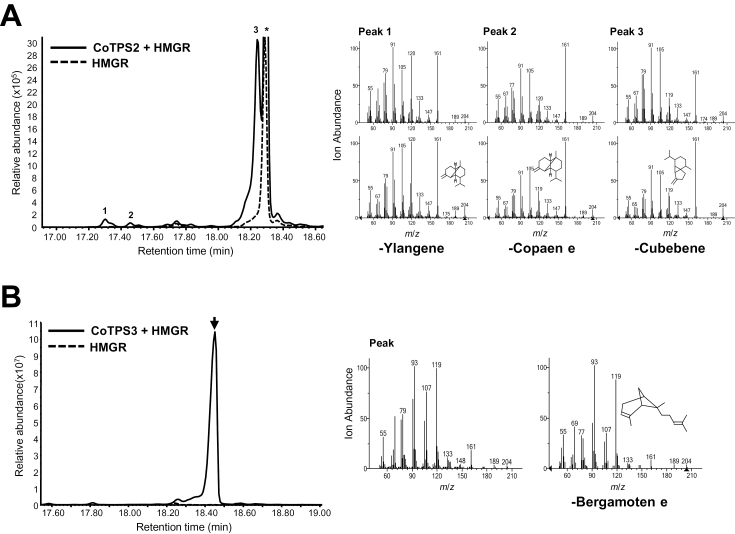
*In vivo* characterization of CoTPS2 and CoTPS3. YFP-fused CoTPS2 (A) or CoTPS3 (B) with or without HMGR was transiently expressed in *N. benthamiana* leaves by *Agrobacterium*-mediated infiltration. The compounds were analysed at 3 d post-infiltration by GC-MS. Numbered peaks or an arrow in the GC traces were identified by the mass spectra reference library, and the mass spectra of compounds are shown on the right side. The expression of HMGR alone in each figure was used as a control. The asterisk indicates a non-specific peak derived from the expression of HMGR in *N. benthamiana* leaves. *m*/*z*, Mass-to-charge ratio.

We further investigated whether CoTPSs would produce the same terpene products *in vivo* using *Agrobacterium*-mediated transient gene expression in tobacco leaves. The YFP-fused CoTPS1, CoTPS2, CoTPS3, or CoTPS4 was expressed in *N. benthamiana* with or without co-expression of *Arabidopsis* HMGR. HMGR catalyses a rate-limiting step of the MVA pathway and its overexpression is known to increases heterologous sesquiterpene production (Song *et al.*, 2012; Jin *et al.*, 2014). Analysis by GC-MS showed that the *in vivo* results were consistent with those obtained *in vitro*. CoTPS2–YFP clearly produced three compounds, β-ylangene, β-copaene, and β-cubebene, when it was co-expressed with AtHMGR (Fig. 8A), whereas Co-TPS3 produced α-bergamotene when co-expressed with AtHMGR (Fig. 8B). The expression of CoTPS2 or CoTPS3 alone without AtHMGR in *N. benthamiana* did not produce any terpenes, which may be due to limiting amounts of the substrate, FPP (Supplementary Fig. S12, available at *JXB* online). CoTPS1 and CoTPS4 characterized as mono-TPS *in vitro* failed to produce any new peaks *in planta*, suggesting that they might require the co-expression of additional genes, probably a rate-limiting step in the non-MVA pathway. Alternatively, compounds formed by these TPSs might be further metabolized endogenously by the plants.

## Discussion

### Dwarf ylang ylang essential oils are quantitatively dominated by sesquiterpene compounds

Plants use the vibrant colours and VOCs of flowers to attract pollinators. Our analysis on volatile essential oils of dwarf ylang ylang flowers (*C. odorata* var. *fruticosa*) showed that over 90% of the volatile essential oils is composed of sesquiterpenes, such as α-farnesene (31.50%), α-bergamotene (26.79%), germacrene D (13.26%), β-caryophyllene (11.57%), humulene (1.63%), farnesol (0.75%), *trans*-β-farnesene (0.77%), and β-ylangen (0.63%) (Fig. 1, Table 1). In addition, we also detected other groups of aromatic compounds such as benzyl benzoate, benzyl salicylate, and eugenol from the flowers of dwarf ylang ylang, but they constituted,3% (Table 1). This is very different from the composition of essential oils reported previously from ylang ylang flowers originating from Madagascar (Gaydou *et al.*, 1986). In our variety, we found a dominance of α-farnesene (31.50%) which was absent from Madagascar ylang ylang and α-bergamotene (26.79%) one of the main constituents of sandalwood oil (Jones *et al.*, 2011). The differences in the chemical composition of essential oils may be due to differences in genetic background, geographical location, growth conditions, and extraction methods (Benini *et al.*, 2012; Brokl *et al.*, 2013). Fragrant flowers from champak (*Michelia champaca* L.), indian cork (*Millingtonia hortensis* L.), and jasmine (*Jasminum sambac* L.) produce high amounts of four sesquiterpenes, β-caryophyllene, β-bergamotene, α-cubebene, and β-cubebene (Samakradhamrongthai *et al.*, 2009). β-Caryophyllene was the most abundant sesquiterpene in scented flowers of *Alstroemeria* cv. Sweet Laura, which is one of only two scented commercial hybrids (Aros *et al.*, 2012). Therefore, these sesquiterpenes are likely to be important contributors to typical fragrances of these flowers. In addition to α-farnesene, we also detected high levels of β-caryophyllene and α-bergamotene in our ylang ylang variety.

### Detection of *TPS* genes from dwarf ylang ylang by RNA-seq

Our RNA-seq approach provided a rich resource to identify and functionally characterize *TPS* genes from the flowers of dwarf ylang ylang. We found approximately 16 candidate *TPS* transcripts for various mono- and sesquiterpenes from the transcriptome data of dwarf ylang ylang flowers. However, many of the candidate *TPS* transcripts contained partial mRNA sequences from our RNA-seq data. Among the four *CoTPS* transcripts studied, the expression level of *CoTPS1* was the highest, followed by *CoTPS2* and *CoTPS3*, with *CoTPS4* being the lowest. However, the RNA-seq expression level of *CoTPS* transcripts did not correlate exactly with the abundance of terpenes produced by these TPSs when analysed by GC-MS. This might be due to post-translational modifications or may be a reflection of different enzyme activities.

### Sequence characteristics of CoTPSs

According to our phylogenetic analysis, CoTPS1 and CoTPS3 were grouped in the TPS-b subfamily, which commonly represents angiosperm mono-TPSs. Generally, the TPS-b group contains two distinct structural domains, the plastid Tp domain and the R(R)X_8_W motif for monoterpene cyclization located in N-terminal region of mature TPS (Bohlmann *et al.*, 1998). CoTPS1 appears to be a typical member of the TPS-b subfamily, and has both these mono-TPS characteristics. *In vitro* studies also showed that CoTPS1 catalysed the formation of a mixture of monoterpenes, β-thujene, sabinene, β-pinene and α-terpinene from GPP. In contrast to CoTPS1, CoTPS3 had a conserved R(R)X_8_W motif but no Tp sequence for plastid targeting, which explained its cytosolic localization in transient expression assays (Fig. 5). Moreover, CoTPS3 used FPP to synthesize the sesquiterpene α-bergamotene *in vitro* and produced the same product *in vivo* (Figs 7C and 8B). Hence, it is a unique sesqui-TPS that not only contains the monoterpene characteristic R(R)X_8_W but also belongs to the TPS-b subfamily associated with mono-TPSs. The protein encoded by *CoTPS3* had a low level of sequence identity of 45–47% to the α-terpineol synthase and *trans*-ocimene synthase from magnolia flower and mountain pepper, respectively, which are mono-TPSs most similar to CoTPS3 (Lee and Chappell, 2008; Chang and Chu, 2011). Similar sesqui-TPSs that reside in the TPS-b phylogenetic clade have been reported in tomato (*Solanum lycopersicum*) and sandalwood (*Santalum album*) (Falara *et al.*, 2011; Jones *et al.*, 2011). Conversely, the CoTPS4 contained the N-terminal 35 aa of a putative plastid Tp sequence but lacked the R(R)X_8_W motif, a characteristic feature of TPS-b mono-TPSs. CoTPS4 was annotated as a plastid geraniol synthase by BlastX analysis, since it closely resembled the geraniol synthase from Madagascar periwinkle, with an amino acid sequence identity of 84% (Simkin *et al.*, 2013). As expected, this protein catalysed the synthesis of geraniol from GPP *in vitro*. Since the new TPS-g family lacking the R(R)X_8_W motif was first defined from snapdragon *TPS* genes related to floral scent biosynthesis (Dudareva *et al.*, 2003), additional *TPS* genes belonging to the TPS-g family have subsequently been identified from *Arabidopsis*, rice, and kiwifruit. These TPSs produce acyclic terpenes, linalool and (*E*)-nerolidol (Yuan *et al.*, 2008; Chen *et al.*, 2011; Green *et al.*, 2012), which eventually became a prominent feature for members of the TPS-g group. As expected from a member of the TPS-g family, CoTPS4 produced an acyclic geraniol, and the protein sequence clustered closely with the grapevine geraniol synthases of the TPS-g subfamily (Martin *et al.*, 2010), indicating that these TPS functions are highly conserved among plants.

### CoTPS2 is a multifunctional and novel sesqui-TPS

Many TPSs are known to synthesize several products simultaneously. Typical multiproduct mono-TPSs such as cineole synthases, terpinene synthases, terpinolene synthases, bornyl diphosphate synthases, carene synthases, and myrcene synthases additionally produce the same compounds such as sabinene, α/β-pinene (Lücker *et al.*, 2002; Chen *et al.*, 2003; Fäldt *et al.*, 2003; Shimada *et al.*, 2004; Fähnrich *et al.*, 2011). Most of the multiproduct TPSs are likely to synthesize one or two compounds dominantly as major products and others as minor components. Interestingly, CoTPS1 was capable of producing all four monoterpenes β-thujene, sabinene, β-pinene, and α-terpinene at similar levels, and was named β-thujene/sabinene/β-pinene/α-terpinene synthase in this study. Monoterpene thujene, usually referred to as α-thujene, has two double-bond isomers known as β-thujene and sabinene. As all thujene synthases identified catalyse the α form, CoTPS1 possessed the ability to cyclize GPP to β-thujene, found in dwarf ylang ylang flowers.

Some sesqui-TPSs belonging to the TPS-a subfamily from magnolia and kiwifruit preserve the N-terminal R(R)X_8_W motif (Lee and Chappell, 2008; Nieuwenhuizen *et al.*, 2009). This was the case with CoTPS2 in this study. CoTPS2 was able to synthesize three kinds of sesquiterpenes, β-ylangene, β-copaene, and β-cubebene (Figs 7 and 8). Similar to CoTPS2, many sesqui-TPSs derived from different plant species have been documented to produce multiple products, which normally arise from enantiomers or common intermediates (Munck and Croteau, 1990; Steele *et al.*, 1998; Mercke *et al.*, 1999; Lee and Chappell, 2008).

Almost all the terpenes produced by the four CoTPSs are present in the ylang ylang essential oils composition as shown in Fig. 1 and Table 1, as well from other sources (Gaydou *et al.*, 1986; Brokl *et al.*, 2013). The two undetectable compounds, geraniol and β-copaene, may be produced in extremely small quantities and possibly could be detected by improved analytical technology such as two-dimensional GC-coupled time-of-flight MS. However, it remains to be clarified whether these two compounds are indeed constituents of dwarf ylang ylang floral VOCs.

TPSs for α-bergamotene and geraniol have been reported in other plant species (Lu *et al.*, 2002; Iijima *et al.*, 2004; Landmann *et al.*, 2007). However, exclusive β-ylangene/β-copaene/β-cubebene synthases have not yet been reported. β-Cubebene synthase gene has been identified in *Magnolia grandiflora* (Lee and Chappell, 2008), as *Mg25*, sharing 55% amino acid sequence identity and 72% similarity to CoTPS2. β-Ylangene/β-cubebene or β-copaene/β-cubebene were found as minor peaks out of a total of 52 or 15 sesquiterpenes synthesized in *in vitro* assays in *Abies grandis* or *Medicago truncatula* (Steele *et al.*, 1998; Arimura *et al.*, 2008). Interestingly, a fungal (*Coprinus cinereus*) sesqui-TPS that synthesizes 10 different sesquiterpenes with δ-cadinene and β-copaene as the major products was capable of synthesizing β-ylangene, when the amino acid residues that presumably interact with a conserved Asp in the two metal-binding motifs were mutated (Lopez-Gallego *et al.*, 2010). β-Ylangene is a diastereomer of β-copaene; however, it was not produced by the wild-type sesqui-TPS (Lopez-Gallego *et al.*, 2010). In conclusion, CoTPS2 is a multifunctional and novel TPS producing three sesquiterpenes, β-ylangene, β-copaene, and β-cubebene, *in vitro* as well as *in vivo*. α-Copaene, the isomer of β-copaene, is a potent attractant for an agricultural pest, Mediterranean fruit flies, *Ceratitis capitata* (Nishida *et al.*, 2000). Future work should address the ecological role of these terpenes in environmental adaptation.

## Supplementary data

Supplementary data are available at *JXB* online.

Supplementary Fig. S1. Compositional variation of dwarf ylang ylang essential oils during flower development.

Supplementary Fig. S2. Total ion chromatograms of essential oils from dwarf ylang ylang flowers.

Supplementary Fig. S3. Variation of four major terpenes during flower development.

Supplementary Fig. S4. Total ion chromatograms of essential oils from dwarf ylang ylang flowers sampled at day and night.

Supplementary Fig. S5. Quality of deep sequencing.

Supplementary Fig. S6. Alignment of deduced amino acid sequences of representative genes involved in biosynthetic pathways for VOCs.

Supplementary Fig. S7. Phylogenetic analysis of DXSs from dwarf ylang ylang.

Supplementary Fig. S8. Comparison of deduced amino acid sequences of four CoDXS small gene family.

Supplementary Fig. S9. Alignment of deduced amino acid sequences of CoTPSs and other plant TPSs.

Supplementary Fig. S10. SDS-PAGE gel showing purified recombinant 6His-tagged CoTPS1, CoTPS2, CoTPS3, and CoTPS4 proteins.

Supplementary Fig. S11. *In vitro* enzymatic assay of recombinant 6His-tagged CoTPS4s using GPP.

Supplementary Fig. S12. Transient expression of CoTPS2-YFP or YFP in *N. benthamiana.*

Supplementary Table S1. Oligonucleotide primers used in this study.

Supplementary Data

## Abbreviations:

CaMV: cauliflower mosaic virus
FPP: farnesyl pyrophosphate
GC-MS: gas chromatography/mass spectrometry
GO: Gene Ontology
GPP: geranyl pyrophosphate
HMGR: 3-hydroxy-3-methylglutaryl-CoA reductase
MEP: 2-C-methyl-d-erythritol 4-phosphate
MVA: mevalonate
ORF: open reading frame
qRT-PCR: quantitative real-time reverse transcription PCR
RI: retention index
RNA-seq: RNA sequencing
Tp: transit peptide
TPS: terpene synthase
VOC: volatile organic compound
YFP: yellow fluorescent protein.
