# Supplementary material for: The floral transcriptome of ylang ylang (Cananga odorata var. fruticosa) uncovers biosynthetic pathways for volatile organic compounds and a multifunctional and novel sesquiterpene synthase
Source: J Exp Bot. 2015 May 8;66(13):3959–75. doi: 10.1093/jxb/erv196 (PMC4473991; doi:10.1093/jxb/erv196)
Supplement: Supplementary Data [file supp_66_13_3959__index.html]

The floral transcriptome of ylang ylang (Cananga odorata var. fruticosa) uncovers biosynthetic pathways for volatile organic compounds and a multifunctional and novel sesquiterpene synthase — The floral transcriptome of ylang ylang (Cananga odorata var. fruticosa) uncovers biosynthetic pathways for volatile organic compounds and a multifunctional and novel sesquiterpene synthase — Supplementary Data 

# The floral transcriptome of ylang ylang (*Cananga odorata* var. *fruticosa*) uncovers biosynthetic pathways for volatile organic compounds and a multifunctional and novel sesquiterpene synthase

## Supplementary Data

Data files

**Files in this Data Supplement:**

- Supplementary Data - Supplementary Data
