# Supplementary material for: The floral transcriptome of ylang ylang (Cananga odorata var. fruticosa) uncovers biosynthetic pathways for volatile organic compounds and a multifunctional and novel sesquiterpene synthase
Source: J Exp Bot. 2015 May 8;66(13):3959–75. doi: 10.1093/jxb/erv196 (PMC4473991; doi:10.1093/jxb/erv196)
Supplement: Supplementary Data [file supp_erv196_jexbot147470_file001.pdf]

Supplementary Figures

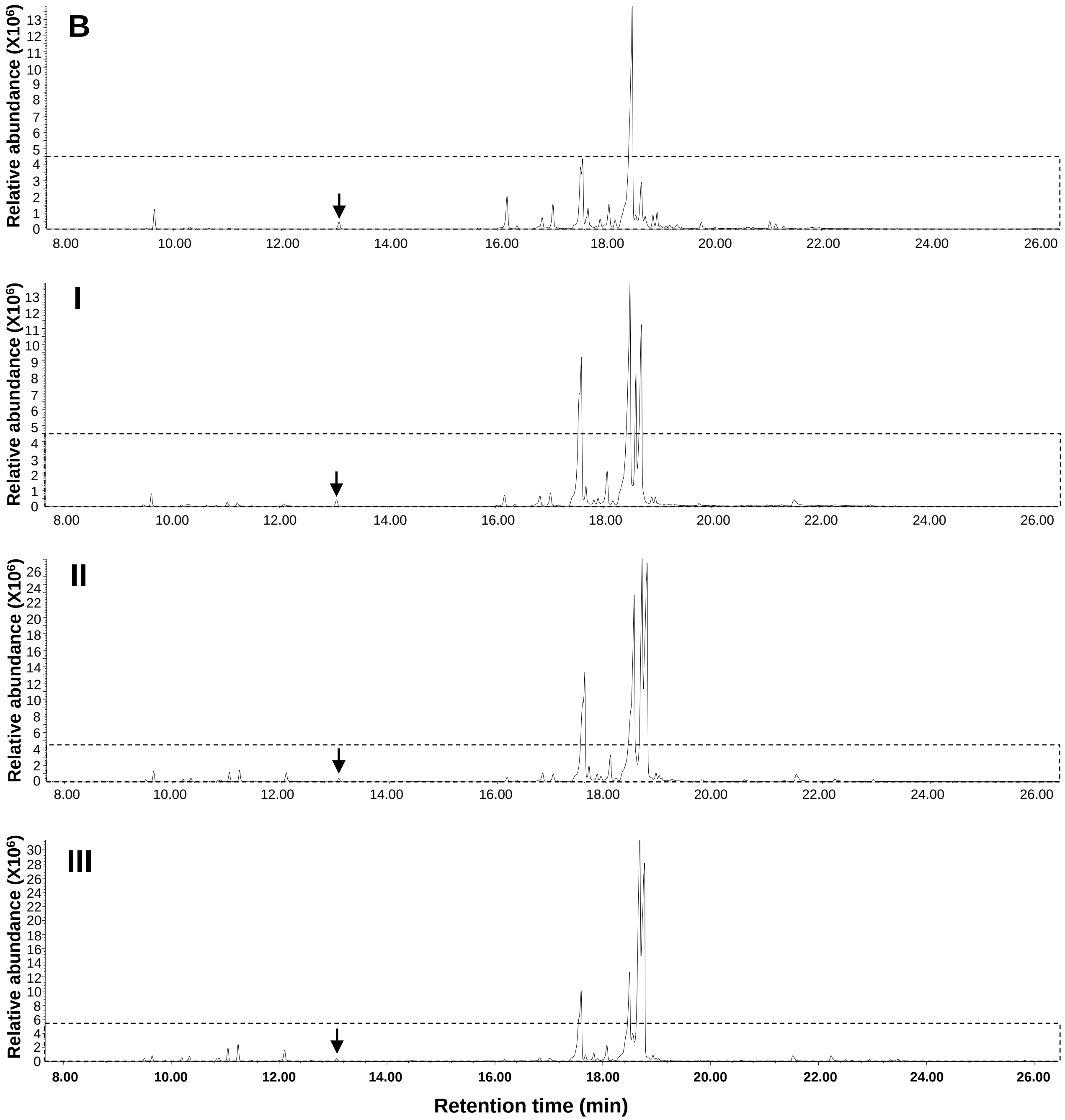

Supplementary Fig. S1. Compositional variation of dwarf ylang ylang essential oils during flower development. B, floral buds; I, undeveloped small flowers; II, mature green flowers; III, fully mature yellow flowers. The arrows indicate the peak of camphor, the internal standard used in the assay.

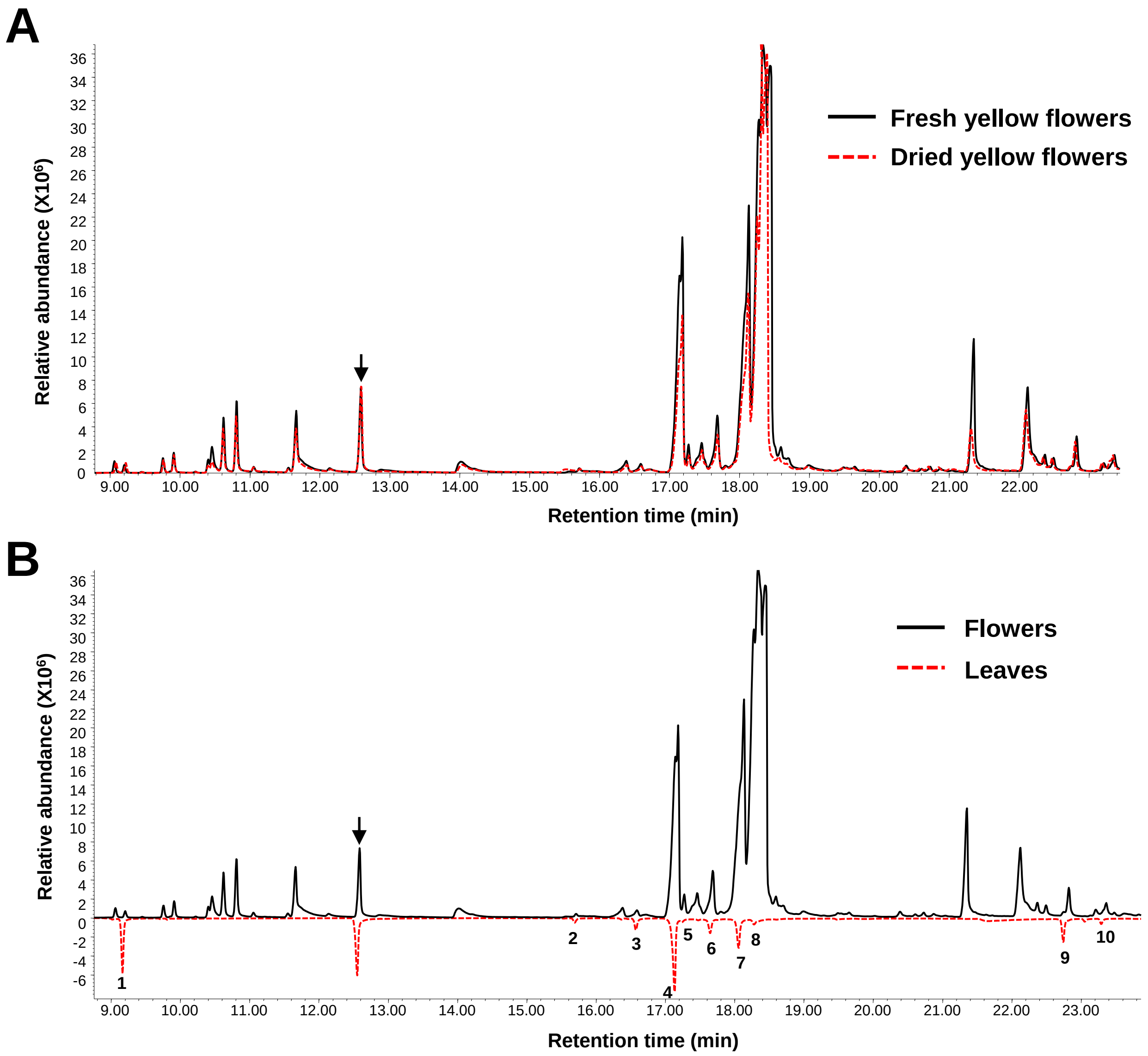

Supplementary Fig. S2. Total ion chromatograms of essential oils from dwarf ylang ylang flowers. (A) GC traces showing no difference between fresh and dried flowers, (B) GC traces of flowers and leaves from dwarf ylang ylang. 1, α-pinene; 2, Elixene; 3, β-elemene; 4, β-caryophyllene; 5, γ-muurolene; 6, Humulene; 7, Germacrene D; 8, γ-elemene; 9 and 10, phytol.
The arrows indicate the peak of camphor, the internal standard used in the assay.

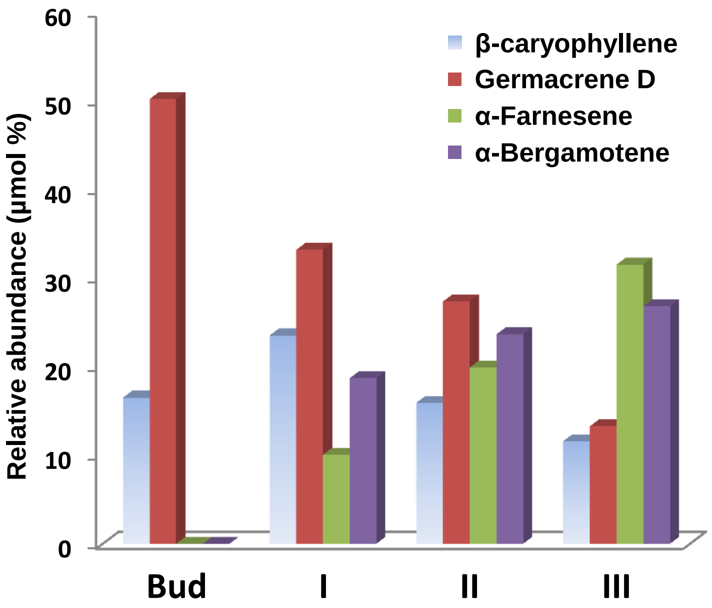

Supplementary Fig. S3. Variation of four major terpenes during flower development. Bud, floral buds; I, undeveloped small flowers; II, mature green flowers; III, fully mature yellow flowers.

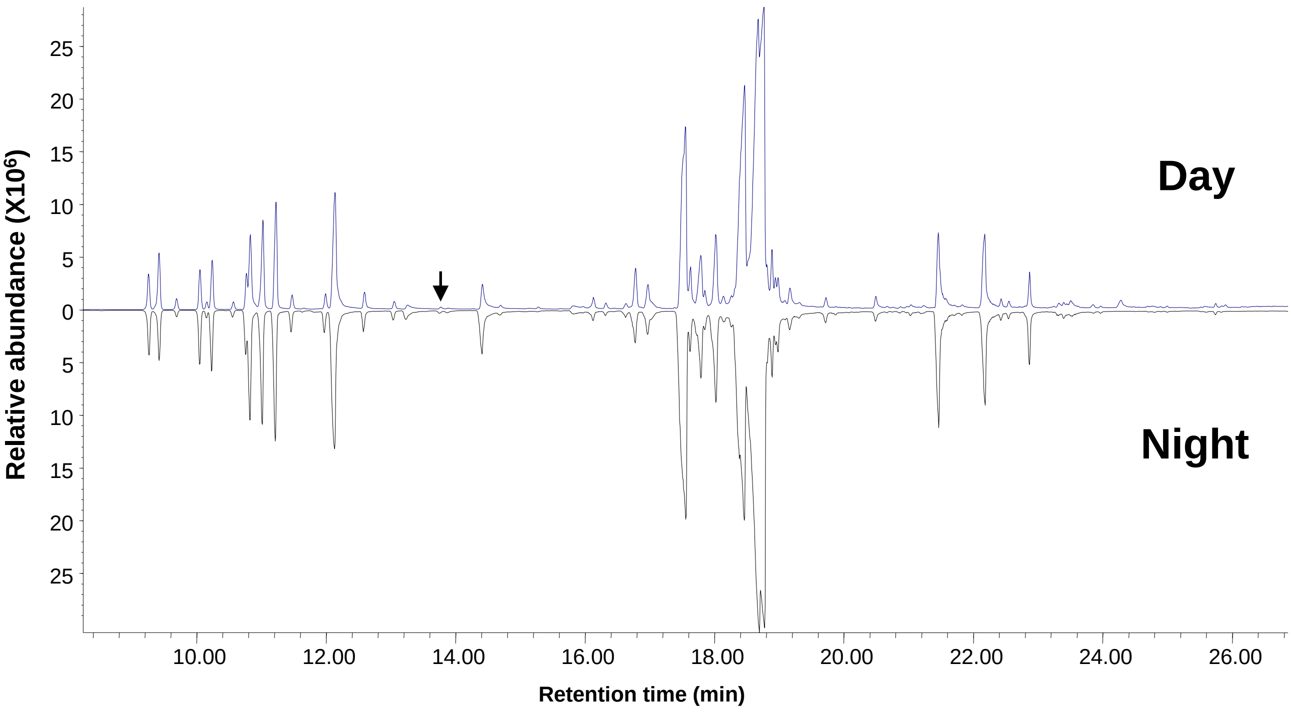

Supplementary Fig. S4. Total ion chromatograms of essential oils from dwarf ylang ylang mature yellow flowers sampled at day and night. The arrows indicate the peak of camphor, the internal standard used in the assay.

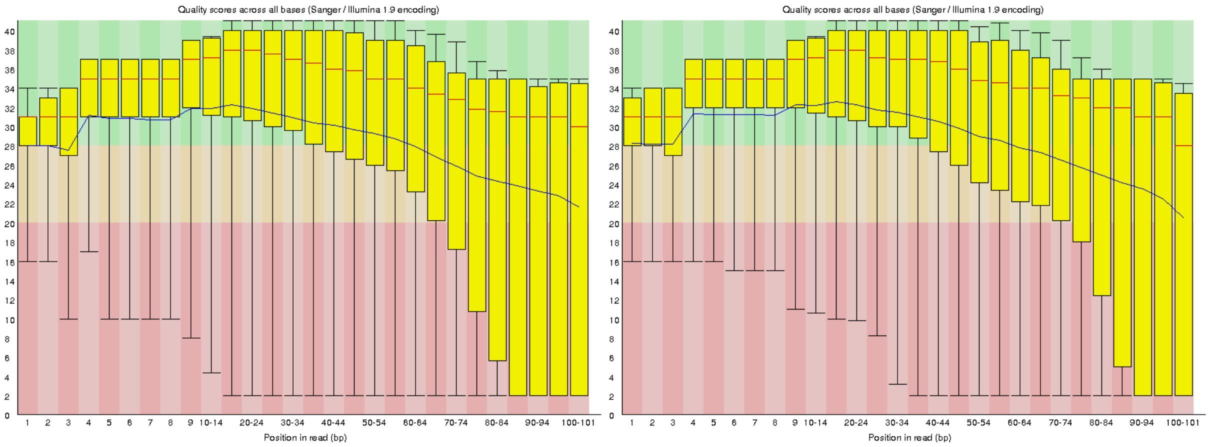

Supplementary Fig. S5. Quality of deep sequencing. The sequence quality was evaluated by FastQC.

A

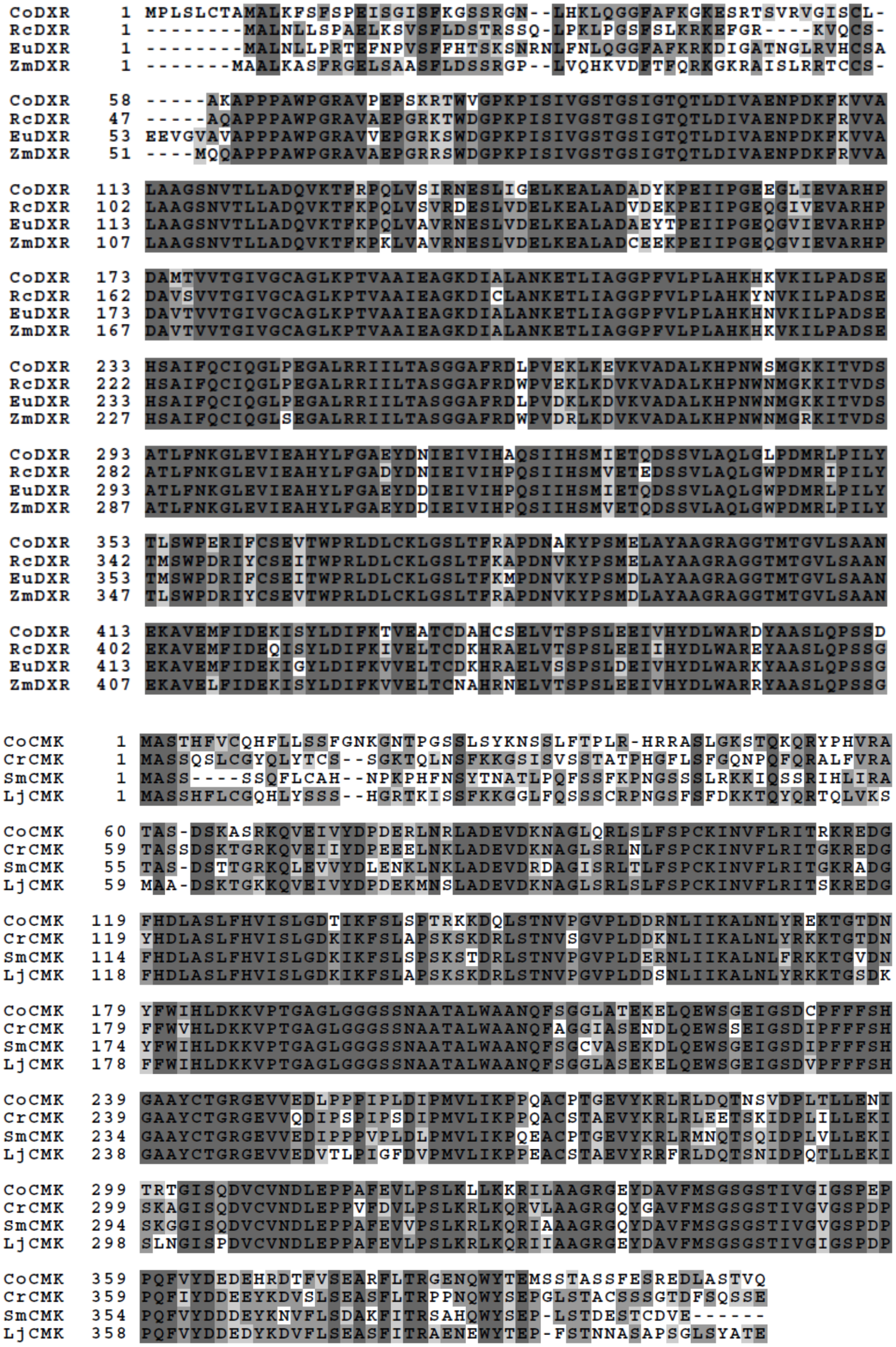

Supplementary Fig. S6. Alignment of deduced amino acid sequences of representative genes involved in biosynthetic pathways for VOCs. (A) Alignment of deduced amino acid sequences of two representative genes, DXR and CMK, involved in MEP pathway. DXR, 1-deoxy-D-xylulose 5-phosphate reductoisomerase; CMK, 4-(cytidine 5'-diphospho)-2-C-methyl-D-erythritol kinase. Co, *Cananga odorata*; Cr, *Catharanthus roseus*; Eu, Eucommia ulmoides; Lj, *Lonicera japonica*; Rc, Ricinus communis; Sm, *Salvia miltiorrhiz; Zm, Zea mays.* Accession numbers: RcDXR (XP_002511399), EuDXR (AFU93070), ZmDXR (NP_001105139), CrCMK (ABI35992), SmCMK (ABP96842), LjCMK (AGE10581). Completely conserved residues are shaded in dark gray, identical residues are shaded in gray, and similar residues are shaded in light gray. Dashes indicate gaps introduced to maximize sequence alignment.

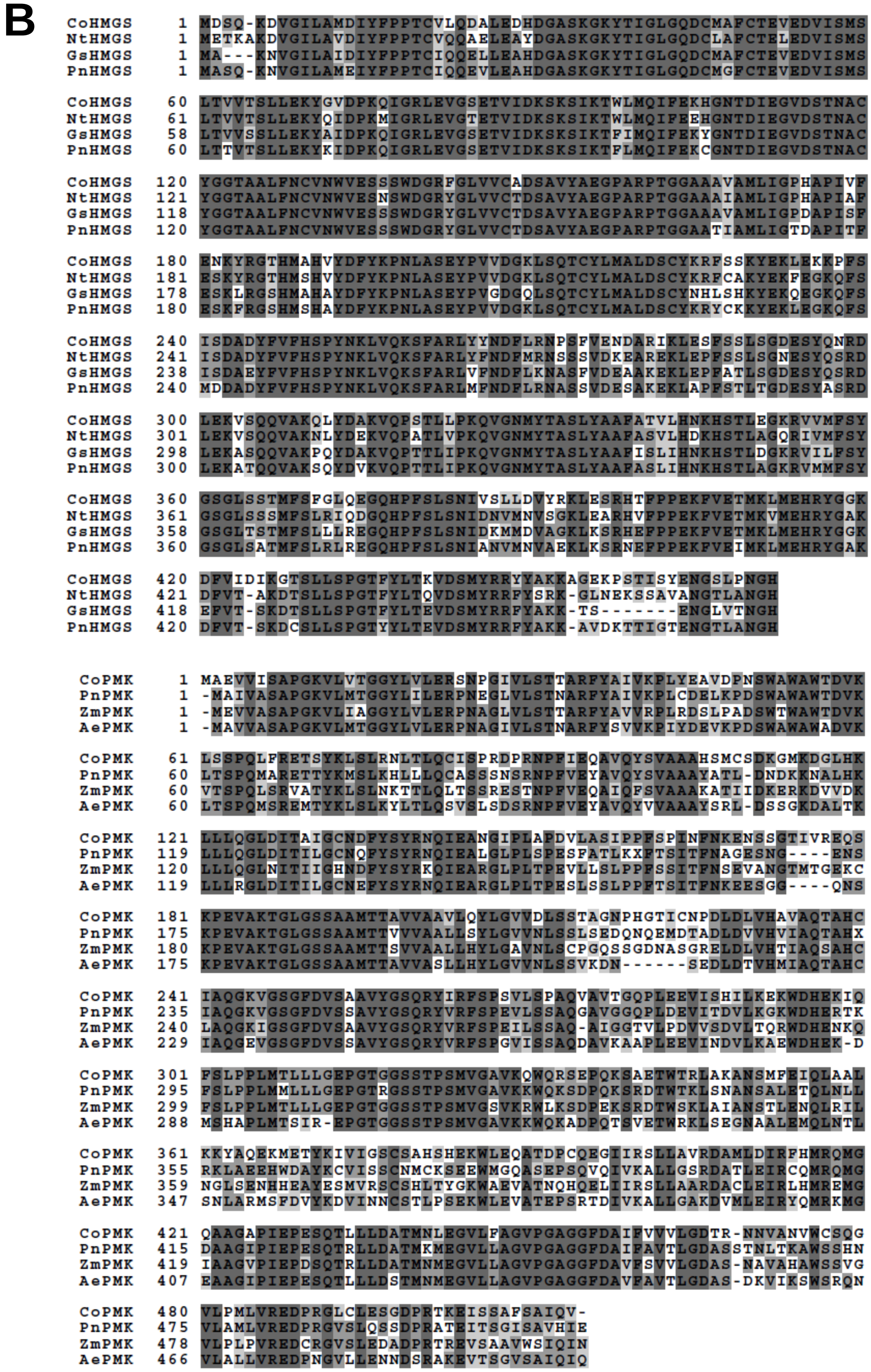

Supplementary Fig. S6 (Continued). (B) Alignment of deduced amino acid sequences of two representative genes, HMGS and PMK, involved in MVA pathway. HMGS, hydroxymethylglutaryl-CoA synthase; PMK, phosphomevalonate kinase. Ae, *Arnebia euchroma*; Co, *Cananga odorata*; Gs, *Glycine soja*; Nt, *Narcissus tazetta*; Pn, *Panax notoginseng*; Zm, *Zea mays*. Accession numbers: NtHMGS (AHF81872), GsHMGS (KHN14128), PnHMGS (AIK21781), PnPMK (AIK21784), ZmPMK (NP_001149345), AePMK (ABY27562). Completely conserved residues are shaded in dark gray, and identical residues are shaded in gray, and similar residues are shaded in light gray. Dashes indicate gaps introduced to maximize sequence alignment.

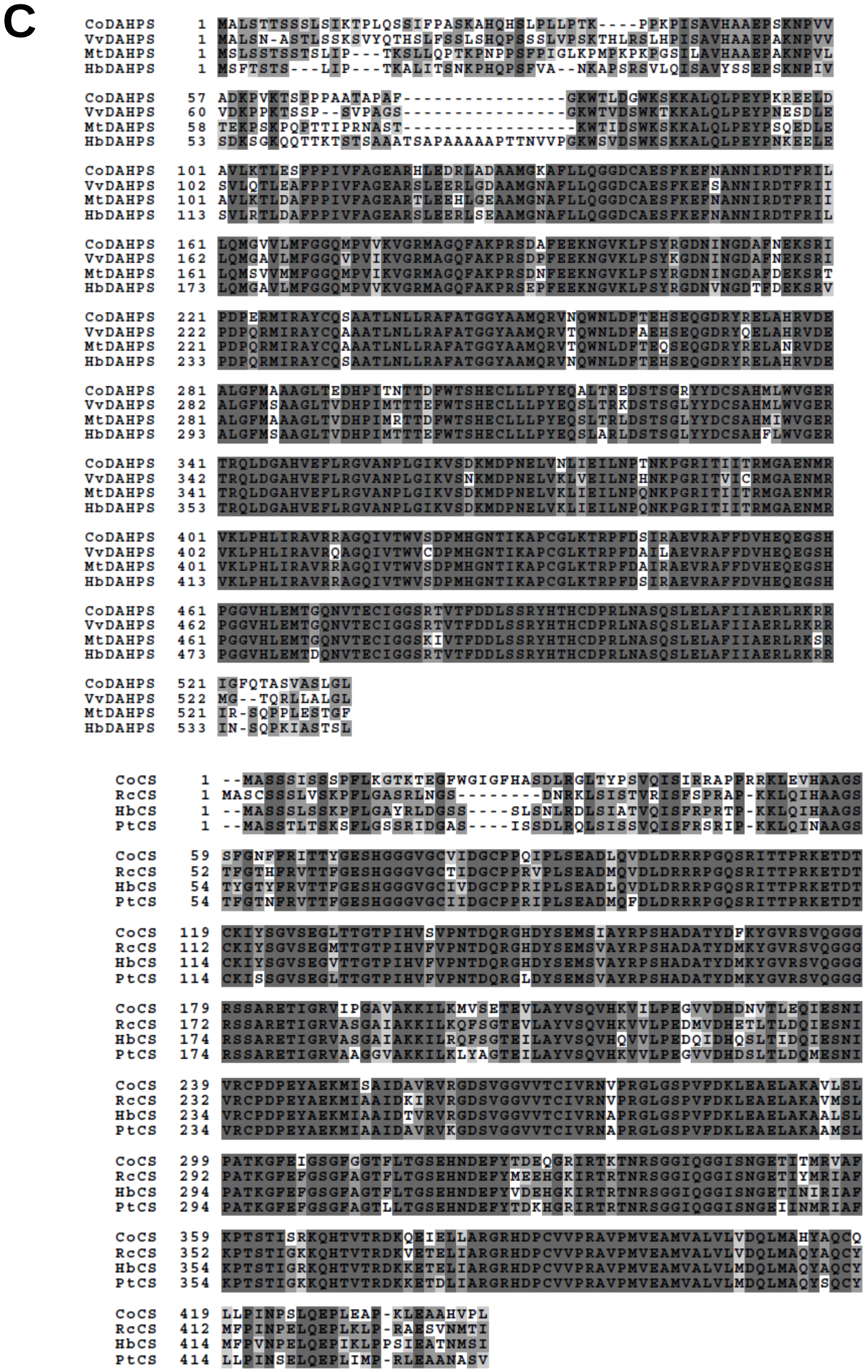

Supplementary Fig. S6 (Continued). (C) Alignment of deduced amino acid sequences of two representative genes, DAHPS and CS, involved in shikimate pathway. DAHPS, 3-deoxy-D-arabino-heptulosonate-7-phosphate synthase; CS, chorismate synthase. Co, *Cananga odorata*; Hb, *Hevea brasiliensis*; Mt, *Medicago truncatula*; Pt, *Populus trichocarpa*; Rc, *Ricinus communis*; Vv, *Vitis vinifera*. Accession numbers : VvDAHPS (NP_001268127), MtDAHPS (XP_003615152), HbDAHPS (AFY09700), RcCS (XP_002529571), HbCS (ADR70879), PtCS (XP_002315301). Completely conserved residues are shaded in dark gray, and identical residues are shaded in gray, and similar residues are shaded in light gray. Dashes indicate gaps introduced to maximize sequence alignment.

D

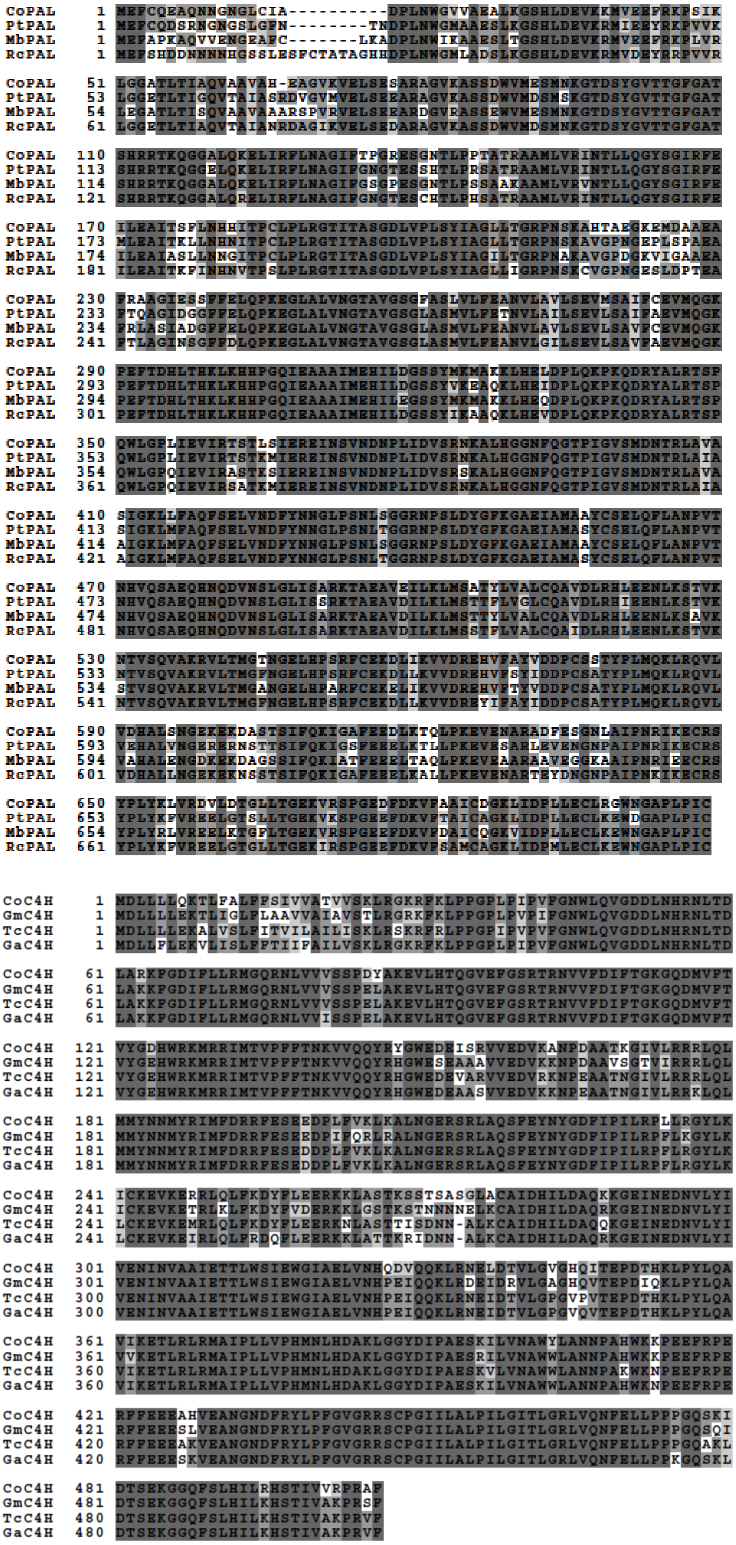

Supplementary Fig. S6 (Continued). (D) Alignment of deduced amino acid sequences of two representative genes, PAL and C4H, involved in phenyl propanoid pathway. PAL, phenylalanine ammonia lyase; C4H, cinnamate-4-hydroxylase. Co, *Cananga odorata*; Ga, *Gossypium arboreum*; Gm, *Glycine max*; Mb, *Musa balbisiana*; Pt, *Populus trichocarpa;* Rc, *Ricinus communis;* Tc, *Theobroma cacao*. Accession numbers : PtPAL (ACC63889), MbPAL (BAG70992), RcPAL (XP_002531677), GmC4H (ACR44227), TcC4H (XP_007011365), GaC4H (AAG10197). Completely conserved residues are shaded in dark gray, and identical residues are shaded in gray, and similar residues are shaded in light gray. Dashes indicate gaps introduced to maximize sequence alignment.

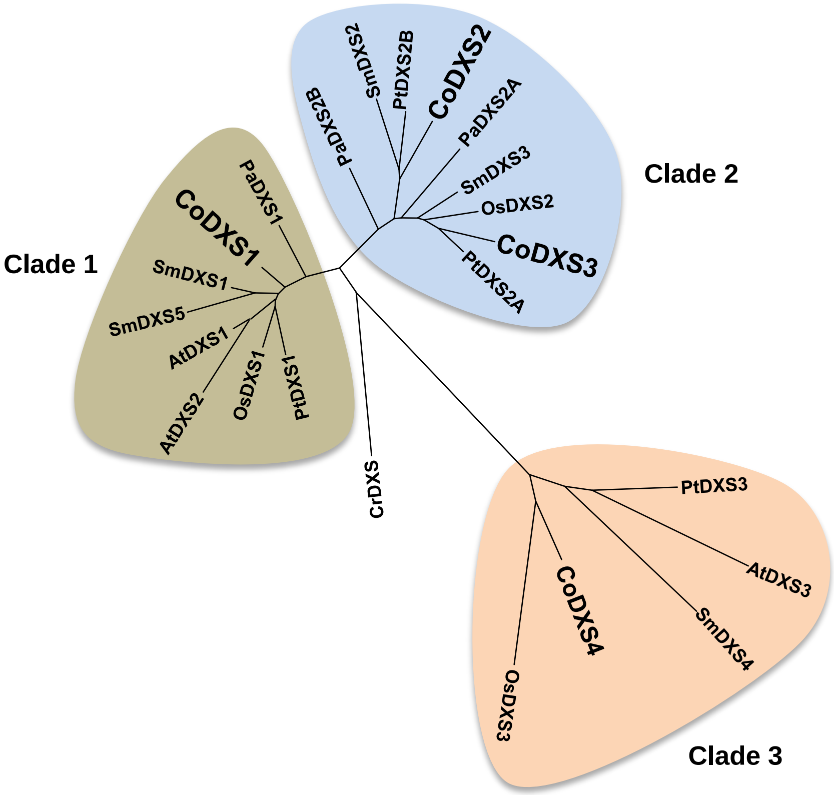

Supplementary Fig. S7. Phylogenetic analysis of DXSs from dwarf ylang ylang. The maximum likelihood tree was drawn by MEGA 6 program from an alignment of full-length CoDXSs (1-deoxy-D-xylulose 5- phosphate synthase) with other plant DXSs. At, *Arabidopsis thaliana*; Os, *Oryza sativa*; Pa, *Picea abies*; Pt, *Populus trichocarpa*; Sm, *Salvia miltiorrhiza*.

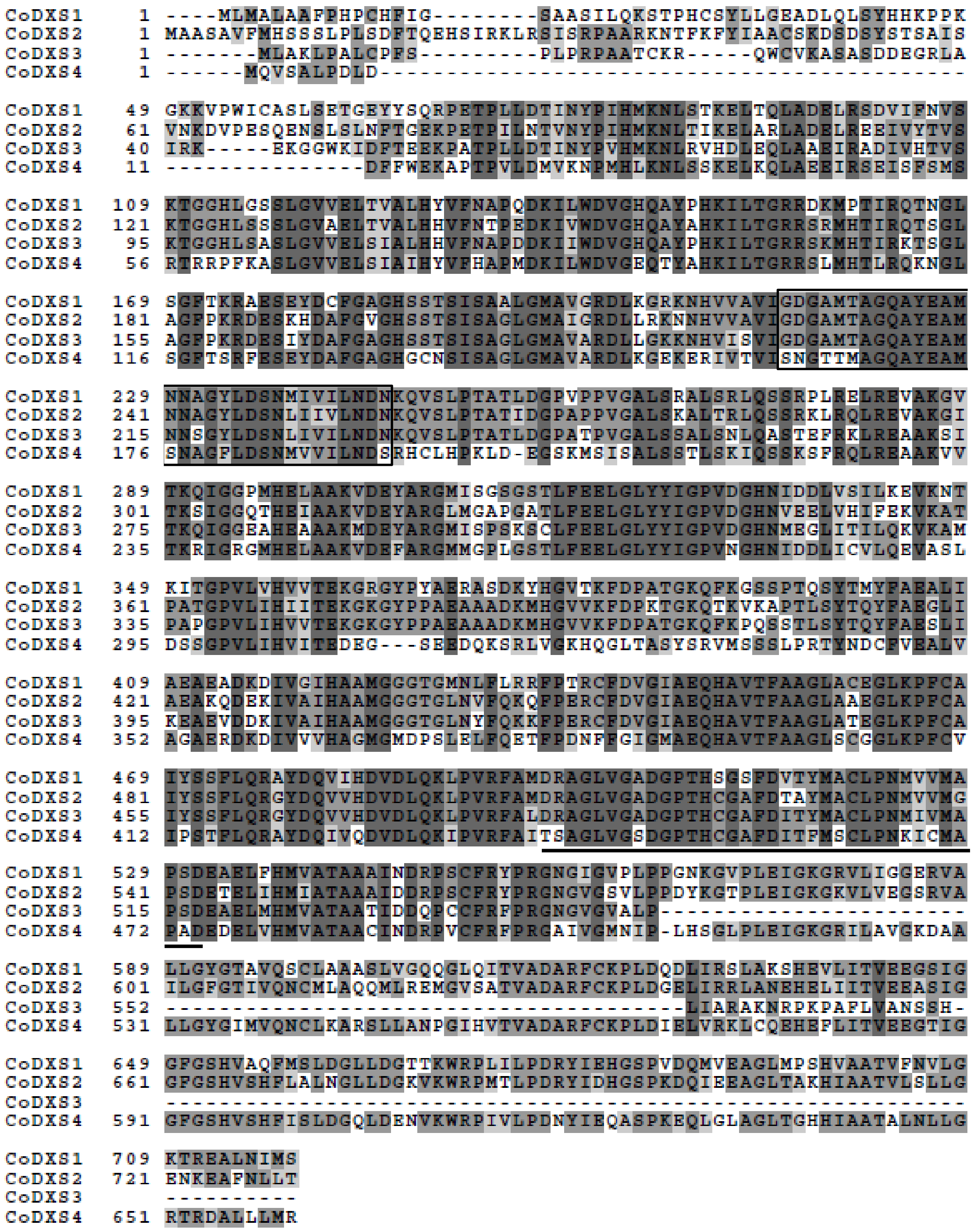

Supplementary Fig. S8. Comparison of deduced amino acid sequences of four CoDXS small gene family. The thiamin diphosphate-binding site and the pyridine binding DRAG Domain are indicated by the open box and the horizontal line, respectively. Completely conserved residues are shaded in dark gray, and identical residues are shaded in gray, and similar residues are shaded in light gray. Dashes indicate gaps introduced to maximize sequence alignment.

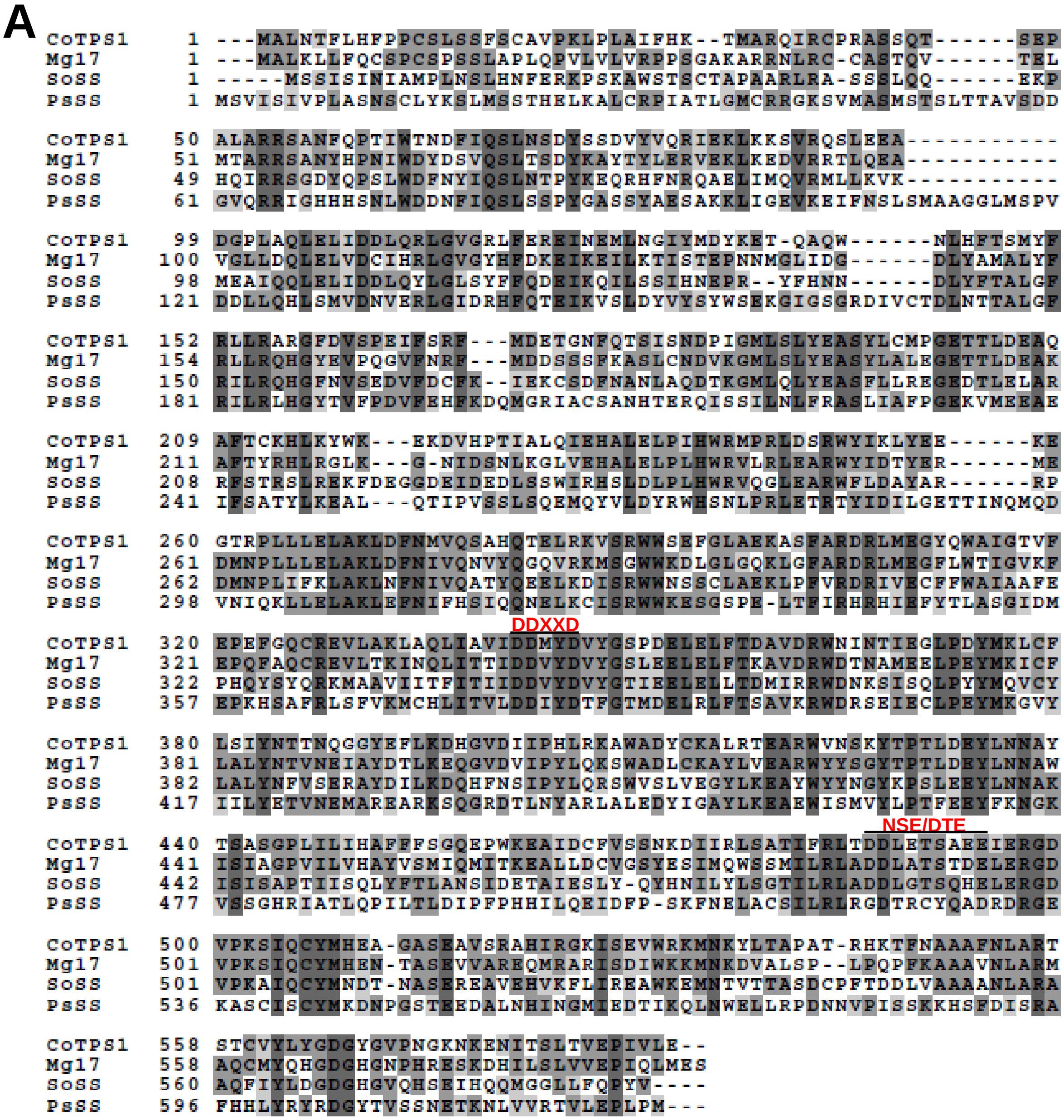

Supplementary Fig. S9. Alignment of deduced amino acid sequences of CoTPSs and other plant TPSs. (A) Alignment of deduced amino acid sequences of CoTPS1 and other plant TPSs. PsSS, *Picea sitchensis* sabinene synthase (ADU85930); SoSS, *Salvia officinalis* sabinene synthase (AAC26018); Mg17, *Magnolia grandiflora* α-terpineol synthase (B3TPQ7). The conserved motifs DDXXD and NSE/DTE are marked. Completely conserved residues are shaded in dark gray, and identical residues are shaded in gray, and similar residues are shaded in light gray. Dashes indicate gaps introduced to maximize sequence alignment.

B

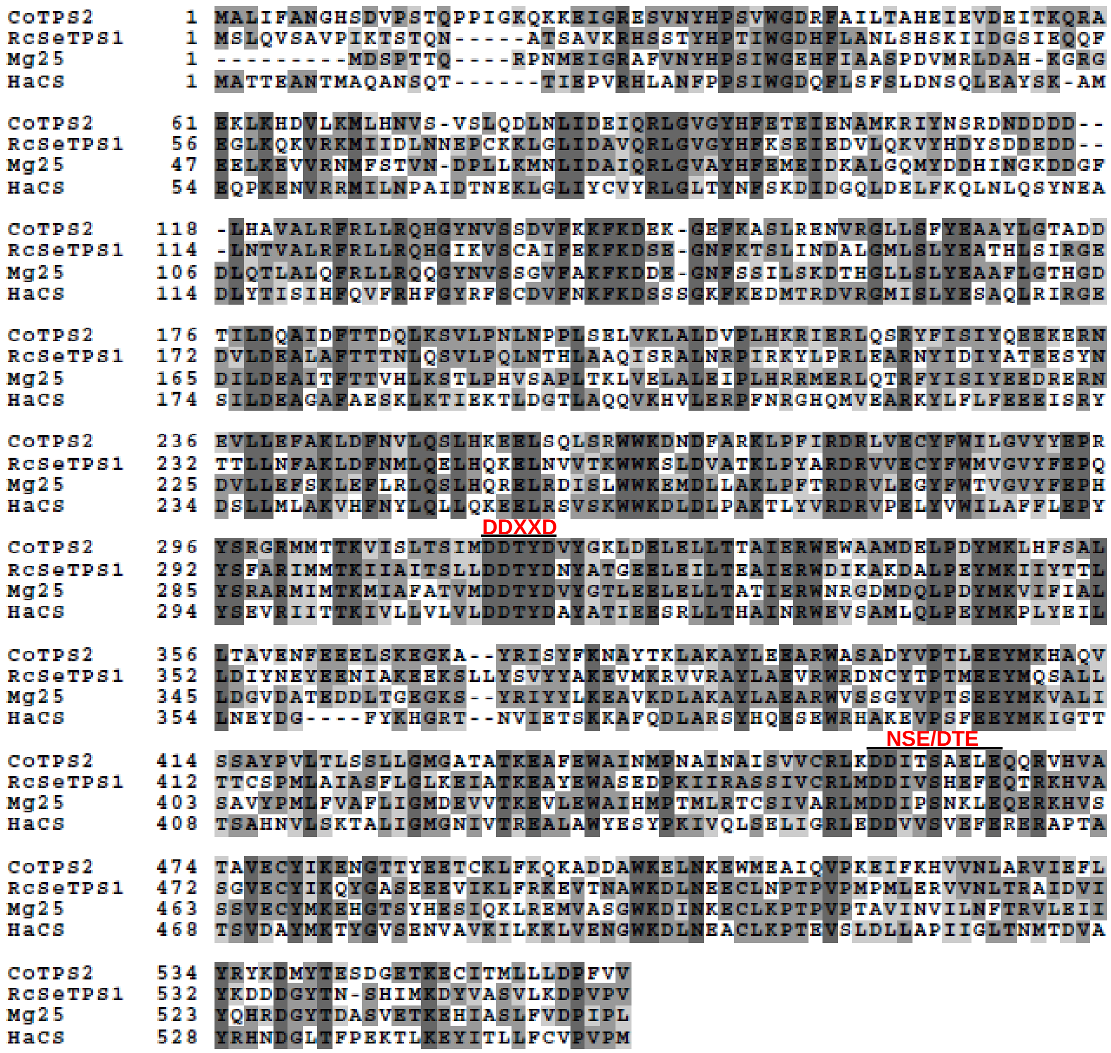

Supplementary Fig. S9 (Continued). (B) Alignment of deduced amino acid sequences of CoTPS2 and other plant TPSs. RcSeTPS1, *Ricinus communis* α-copaene synthase (B9S9Z3); Mg25, *Magnolia grandiflora* β-cubebene synthase (B3TPQ6); HaCS, *Helianthus annuus* α-copaene synthase (Q4U3F6). The conserved motifs DDXXD and NSE/DTE are marked. Completely conserved residues are shaded in dark gray, and identical residues are shaded in gray, and similar residues are shaded in light gray. Dashes indicate gaps introduced to maximize sequence alignment.

C

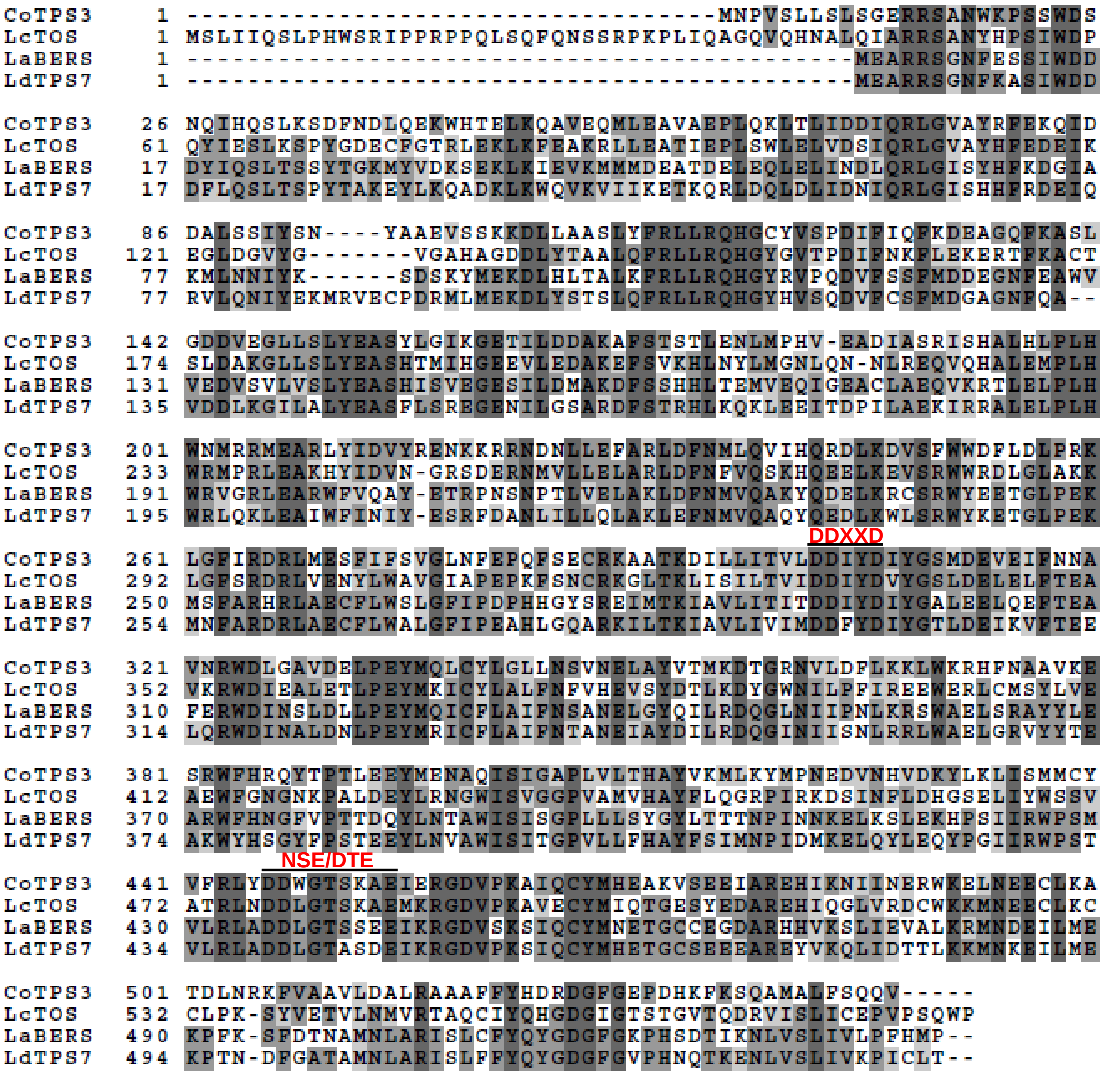

Supplementary Fig. S9 (Continued). (C) Alignment of deduced amino acid sequences of CoTPS3 and other plant TPSs. LcTOS, Litsea cubeba trans-ocimene synthase (AEJ91554); LaBERS, *Lavandula angustifolia* α-bergamotene synthase (Q2XSC4); LdTPS7, *Lippia dulcis* α-bergamotene synthase (J7LQ09). The conserved motifs DDXXD and NSE/DTE are marked. Completely conserved residues are shaded in dark gray, and identical residues are shaded in gray, and similar residues are shaded in light gray. Dashes indicate gaps introduced to maximize sequence alignment.

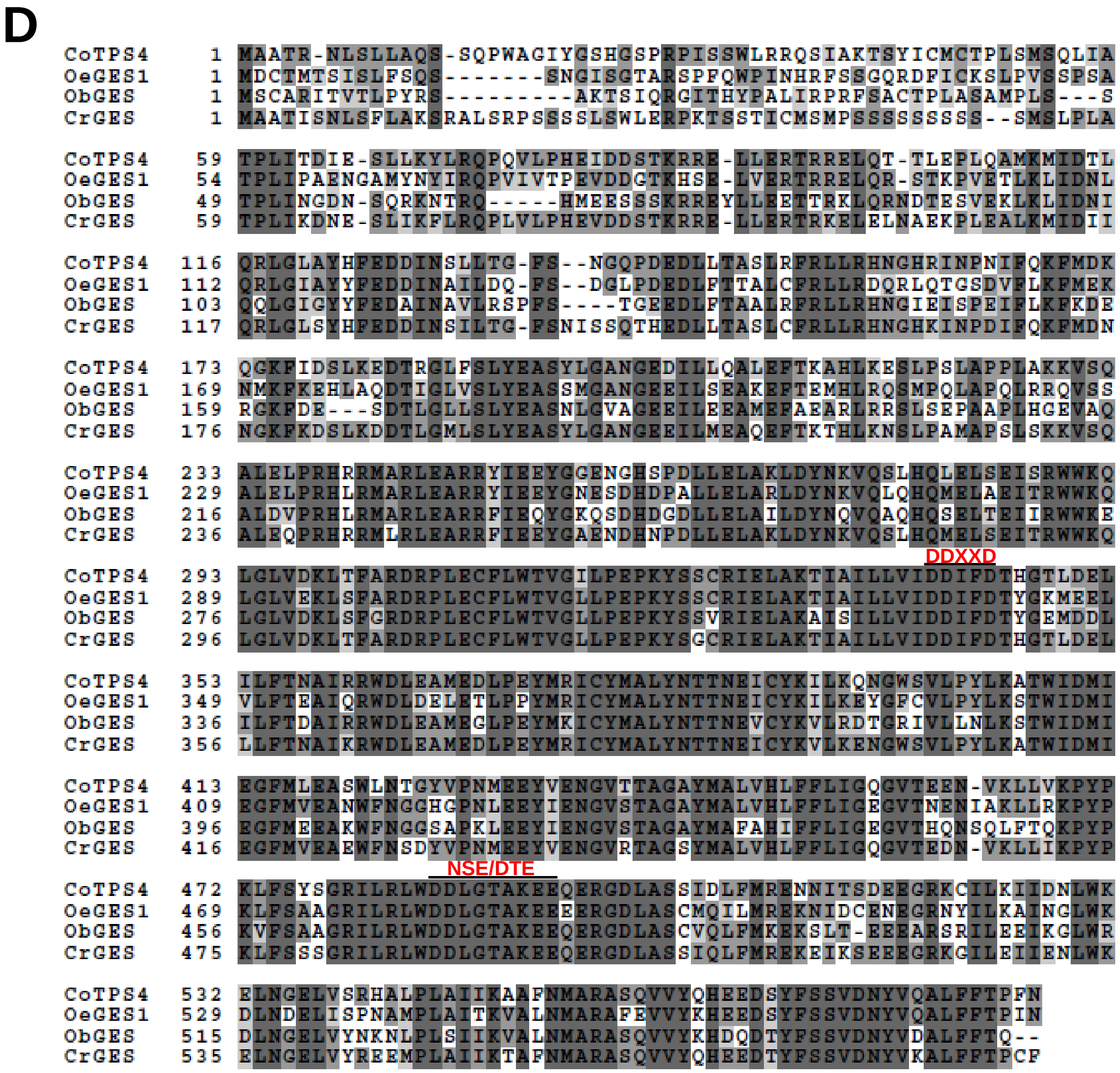

Supplementary Fig. S9 (Continued). (D) Alignment of deduced amino acid sequences of CoTPS4 and other plant TPSs. ObGES, *Ocimum basilicum* geraniol synthase (AAR11765); OeGES1, *Olea europaea* geraniol synthase 1 (AFI47926); CrGES, *Catharanthus roseus* geraniol synthase (AFD64744). The conserved motifs DDXXD and NSE/DTE are marked. Completely conserved residues are shaded in dark gray, and identical residues are shaded in gray, and similar residues are shaded in light gray. Dashes indicate gaps introduced to maximize sequence alignment.

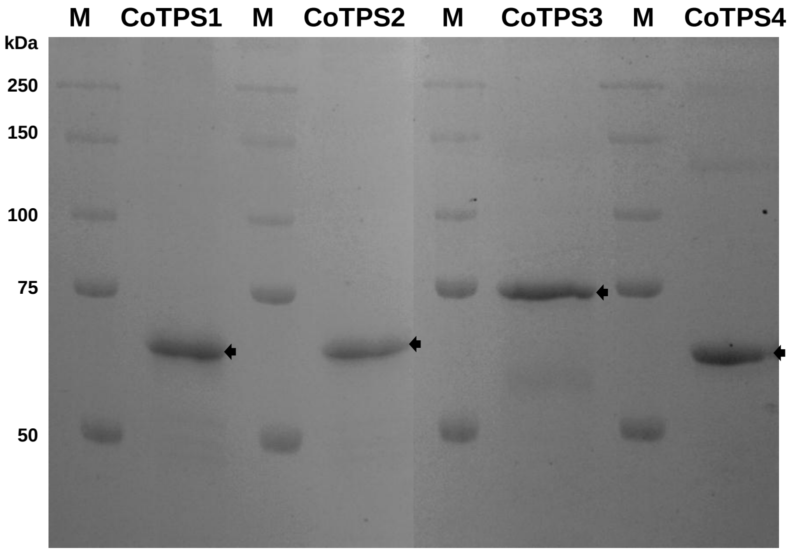

Supplementary Fig. S10. SDS-PAGE gel showing purified recombinant 6His-tagged CoTPS1, CoTPS2, CoTPS3 and CoTPS4 proteins. The position corresponding to the recombinant proteins are indicated by an arrow. M; Precision Plus Protein all blue standards (Bio-rad), kDa; kilodalton.

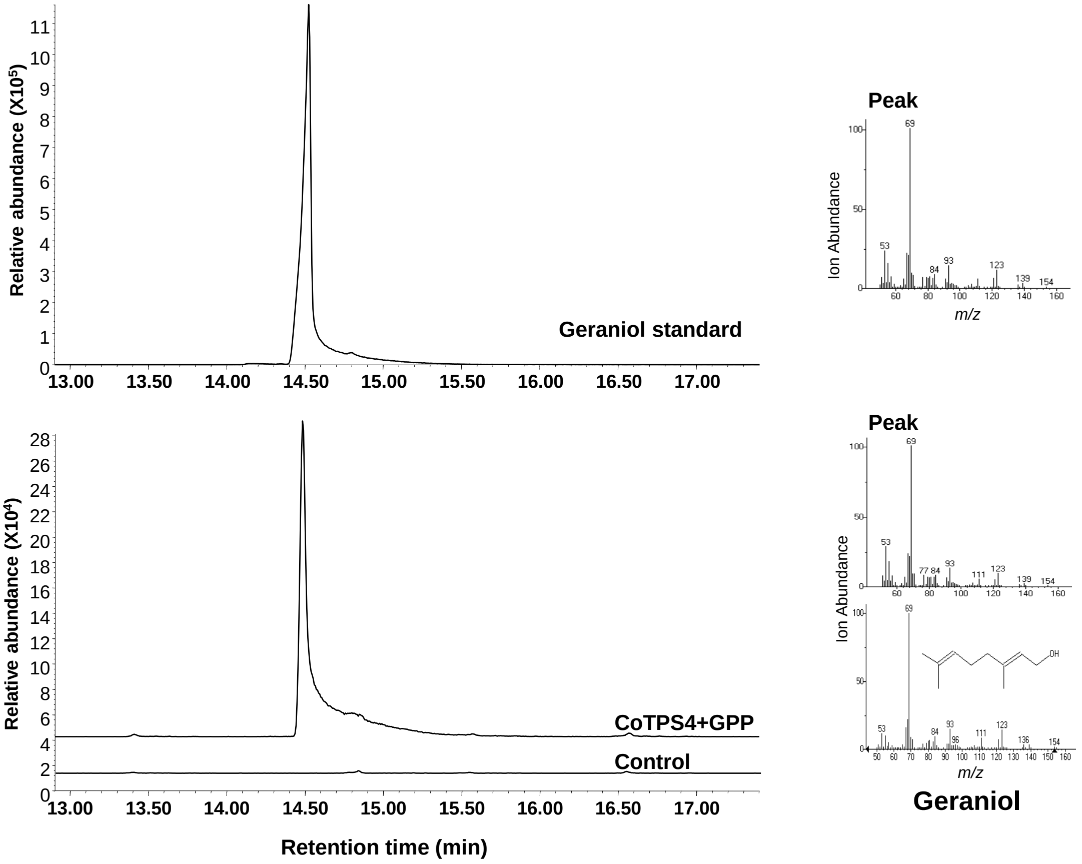

Supplementary Fig. S11. *In vitro* enzymatic assay of recombinant 6His-tagged CoTPS4s using GPP. The reaction products were analysed by GC-MS. The peak was identified by the mass spectra reference library and comparison of retention time with those of authentic standard (geraniol standard). Mass spectra for the geraniol standard and the peak formed with GPP are shown on the right. m/z, mass-to-charge ratio. Control, control assay using a heat-inactivated recombinant protein.

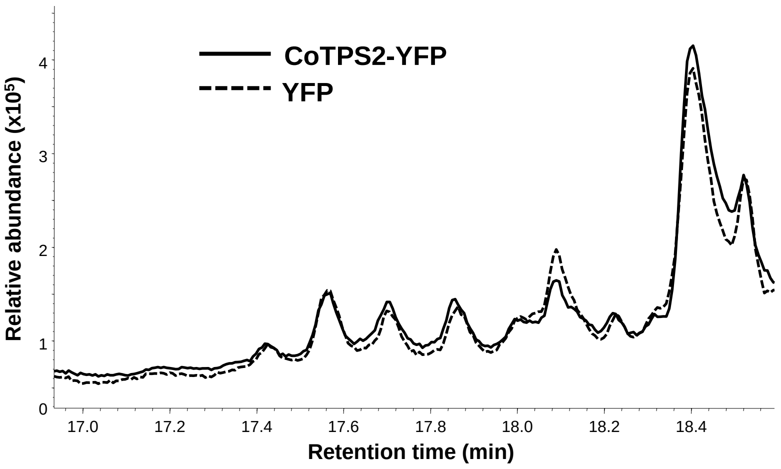

Supplementary Fig. S12. Transient expression of CoTPS2-YFP or YFP in *N. benthamiana.*CoTPS2-YFP or YFP was transiently expressed in *N. benthamiana* leaves by *Agrobacterium*-mediated infiltration. The chemical composition was analysed 3 dpi (days post-infiltration) by GC-MS.

Supplementary Table 1. Oligonucleotide primers used in this study.

Name	Forward	Reverse
**for qRT-PCR**		
DXS1	TTTGATTGGGGGTGAGAGAG	GCCATCAAGGGACATGAACT
DXS2	GGGGATCACAAAATCCATTG	TGGGTAGCCTTTTCCCTTTT
DXS3	CTTCTGCAAGCCCCTAGATG	GTTGCAGCAATGTGCCTAGA
DXS4	AAGGGGAGAATCCTTGCAGT	TGTTCTTGGCAAAGCTTCCT
DXR	GTTCTGAGTGCAGCCAATGA	CAAGAACAGGTCGCAGATCA
CMS	AGGAGCGTGTCAGTGGTTCT	TGGCAAATTTCAGGTCAACA
CMK	TCGGTTCTGATTGTCCCTTC	CGAAGGCGCTTGTAAACTTC
MCS	ACGCAAGCAAAAACTCTCGT	CGGTAGCGTTATCAGCATCA
HDS	GTGGCTCCATTTGAGGAAAA	AATCCACAGTGGCAAGATCC
IDI1	ACTGTACAAGGCCCCATCTG	CCCACCATTTCAGAAGGAAA
IDI2	CATGAGTTGCTCCTTCAGCA	AAGCTTCCTTTGTGCAGCAT
AACT	TGCAGTGAAGTCTGCTGGTC	CAACAGAGTTGCCACACAGC
HMGS	CAAGACTTGGCTGATGCAAA	AACAACAAGCCCAAATCGTC
HMGR	CAGGCTGATTTCCCTGACAT	CTGGCATGTGCATTGTATCC
MVK	GGTGTTTCGGAGAGAGCTTG	ACCCCCATGCACTGAAGTAG
PMK	TGCTCCTCGATGCTACAATG	AGGGTCTTCTCTGACCAGCA
MPDC	CCCATCAATGACAGCATCAG	CGTATTCGGATCTCCCTCAA
DAHPS	ATCCTCCATCTTCCCTGCTT	TTCTTGGATTTCCAGCCATC
DHQS	CCTTTCCTTCCGCTTTCTCT	TTCCATGAACATGCCTTTGA
SDH	TGATCAGCTAGAGCGCAGAA	CCATCTGTTCGACGGATTTT
SK	CACCGTTCCGAAGATTTGTT	CCAGACCCCATCATACCAAC
CS	GCTGCTGGAAGTTCTTTTGG	TCTGTCCTGGCCTCCTTCTA
PAL	AATCTCTCAGGGGGAAGGAA	CAACCCCAGCGAGTTTACAT
SAMT	TTAACGGCAGGAAGAGCCTA	ATCACGGCCTTCATTTCTTG
CHS	TTCTGTTCCTCAAGCGGAGT	CGGAAGTAGCCCACATTCAT
CHI	CATGAGCATGGTCAGGAAGA	GCACAGCAACTCACTTTCCA
F3H	TATCTCTGGTGGGGATCGAC	CTGCTTCTCCCTGAAGATGG
DFR	GCAATGGTGATTGGTCCTCT	GAAGCGGAATTGATGGGTAA
C4H	ACCCACAAGCTTCCCTACCT	AGGTACCAGGCATTCACCAG
4CL	GTTGCACCTGCAGAACTTGA	ACCTTCCGCAGTCTCTTGAA
CCR	GGAGCGATCTGGACTACTGC	GAACATCGACGTAGGCCTGT
CVOMT	GCATGCTCACTCATTCTGGA	CCCAAAGACTTTTTCCGTGA
C3H	ACGCTCCAGGAGAAGTACGA	CACACGGTCCAATTCCTCTT
COMT	ACTCTCCAGTGCCTGTGCTT	AGCTGCCCGTTGTCTAGTGT
CCOMT	TTGGATACCAGCGTCTACCC	CTCACGGTTGATGTCCATTG
CAD	AAAGTGGGAACAACGAATGC	ACCGACCTCTTTGACCACAC
TPS1	AGGGCCTTCCAGATTACATGAAGC	AGCGTTGGAGTGTACTTGCTATTG
TPS2	CATCGATCATGGATGACACATATGATG	CTCAACAGCAGTCAGAAGGGCAC
TPS3	AGGCTGTGGCTGAACCTCTA	AGCAGATCCTTCTTGCTGGA
TPS4	TAGCCATCTTACTGGTGATTGACG	GTAGTGTTGTACAATGCCATGTAAC
Actin	CTGGACGTGACCTCACAGATGCT	TCTTCTCAACAGAGGAGCTGCTCT
**for TOPO cloning**		
TPS1	*CACC***ATG**GCCTTGAATACGTTCTTGCATTTTCC	CTCAAGCACGATGGGTTCAACGGTAAGTGA
TPS2	*CACC***ATG**GCACTTATATTTGCAAATGGCCACTCTGA	ATCAACAAAACGGTCCACTAGCACCATCGCA
TPS3	*CACC***ATG**AATCCTGTTTCTCTTTTGAGCTTATCAGGAG	AACTTGTTGGGAAAATAGAGCCATGGCTTGA
TPS4	*CACC***ATG**GCTGCTACGAGAAACCTTTCTTTACTT	ATTGAAAGGCGTGAAAAACAAAGCTTGCA
**for *E.coli* expression vectors**		
TPS1	aaGGATCCg**ATG**GCCTTGAATACGTTCTTGCATTTTC	aaGCGGCCGCTTACTCAAGCACGATGGGTTCAAC
TPS2	aacGTCGAC**ATG**GCACTTATATTTGCAAATGGCCACTCTGA	aaaGCGGCCGCTTAATCAACAAAACGGTCCACTAGCA
TPS3	aacGGATCCg**ATG**AATCCTGTTTCTCTTTTGAGCTTATCAGGAG	aacGTCGACAACTTGTTGGGAAAATAGAGCCATGGCT
TPS4	aaaGGATCCt**ATG**GCTGCTACGAGAAACCTTTCTTTACTT	aaaGTCGACATTGAAAGGCGTGAAAAACAAAGCTTGCA

GGATCC, *Bam* HI; GCGGCCGC, *Not* I; GTCGAC, *Sal* I
